# Prevalence of Suspected Developmental Delays and Behavioural Problems Identified Using Screening Tools Among Brazilian Children: A Systematic Review and Meta‐Analysis

**DOI:** 10.1111/cch.70379

**Published:** 2026-11-14

**Authors:** Rafaela Cristina Vieira e Souza, Larissa Bueno Ferreira, Tamires Nunes dos Santos, Luana Caroline dos Santos

**Affiliations:** ^1^ Department of Nutrition Federal University of Minas Gerais Belo Horizonte Brazil

**Keywords:** behavioural problems, Brazil, child development, developmental delay, prevalence, screening tools

## Abstract

**Background:**

Developmental delays and behavioural problems in childhood may have important implications for long‐term health, but reliable national estimates in Brazil remain limited. Existing evidence is predominantly derived from regional studies using different screening instruments and outcome definitions, limiting the comparability and generalisability of prevalence estimates.

**Objectives:**

This study aimed to estimate the pooled prevalence of suspected developmental delays and behavioural problems in Brazilian children assessed through screening tools.

**Methods:**

A systematic review and meta‐analysis were conducted following the PRISMA and MOOSE guidelines. Search was performed in Embase, LILACS, PsycINFO, PubMed, Scopus, Web of Science and grey literature, up to September 2025. Observational studies reporting the prevalence of developmental delays and/or behavioural problems in Brazilian children aged 0–10 years, assessed through screening instruments, were included. Random‐effects meta‐analyses were conducted to estimate pooled prevalence, and subgroup analyses per region and instrument were performed.

**Results:**

Fifty‐nine studies from 16 Brazilian states were included. The pooled prevalence estimates of suspected developmental delays reached 28.72% (95% CI: 23.21%–34.95%), whereas behavioural problems identified through screening was 28.17% (95% CI: 22.05%–35.23%). Significant heterogeneity was observed. Suspect developmental delay prevalence ranged from 15.14% in the Midwestern region to 54.65% in the Northern, and behavioural problems ranged from 18.42% in the Northern to 32.10% in the Northeastern. Differences by screening instruments used and region were statistically significant (*p* < 0.01).

**Conclusion:**

The available evidence indicates that suspected developmental delays and behavioural problems are frequently identified among the populations of Brazilian children assessed in the included studies. Nationally representative studies and continuous investments in early developmental surveillance are essential actions to strengthen early detection and intervention strategies in primary healthcare

## Summary

- Pooled estimates from the included studies were 28% for both domains identified through screening tests: suspected developmental delays and behavioural problems.
- Prevalence estimates varied by geographic region and screening instrument.
- Nationally representative studies and culturally appropriate, validated screening tools are needed to strengthen the evidence base and inform child health surveillance.

## 1 Introduction

Child development begins in the intrauterine period, and it encompasses a wide range of interconnected processes, like physical growth, neurological maturation and the progressive acquisition of cognitive, social, behavioural and emotional abilities, including gross and fine motor skills, language and communication, cognitive abilities, problem‐solving, social interaction and adaptive or personal‐social skills. (Sandler et al. 2001; Sigolo and Aiello 2011). In contrast, behavioural problems encompass difficulties in the child's emotional, behavioural and social functioning, which may include emotional symptoms, conduct problems, hyperactivity or inattention, difficulties with peer relationships and challenges in social or adaptive behaviour (Weitzman et al. 2015). Although these constructs are conceptually distinct, they are interrelated and may influence one another throughout childhood.

Despite the multiple efforts to improve child health conditions, the American Academy of Pediatrics (Weitzman et al. 2015) has reported that developmental and behavioural health disorders are among the top five positions in the ranking of chronic paediatric conditions associated with functional impairment. According to data from the US National Survey of Children's Health (Leeb et al. 2024), collected from 2016 to 2021, the lifetime prevalence of mental, behavioural and developmental disorders in children in the age group 3 to 17 years increased from 25.3% to 27.7%. Identifying at‐risk children as early as possible has become a public health priority given the increasing prevalence of developmental and behavioural disorders.

Thus, the most effective approach among strategies used for the early detection of children at risk is the use of standardised screening instruments properly and periodically applied (de Albuquerque and da Cunha 2020; de Lima et al. 2016). Screening is herein understood as running standardised tests designed to practically and systematically detect risks or delays likely influenced by factors ranging from conception to birth, including neurological conditions, malnutrition and environmental, familial and socioeconomic factors (de Lima et al. 2016).

Developmental and behavioural screening should be performed together in clinical practice (Weitzman et al. 2015), because it is a fast assessment procedure aimed at early identifying children who may require diagnostic or intensive evaluation (Sandler et al. 2001). Screening main functions encompass providing ongoing and periodic information on the child's development and behaviour, identifying children who may require more detailed assessments. A positive screening result indicates increased risk or the need for further assessment and should not be interpreted as a clinical diagnosis of a developmental delay or behavioural disorder (Sandler et al. 2001; Weitzman et al. 2015). Earlier identification of at‐risk children is the opportunity to deliver early intervention (Sandler et al. 2001) and to improve the child's overall prognosis (Sigolo and Aiello 2011).

Several instruments to assess developmental and behavioural screening in children are available. However, they must prove themselves valid, reliable and capable of ensuring their appropriateness for use in children and families by representing the cultural, linguistic and economic diversity they were originally developed for (Bliznashka et al. 2025; Sandler et al. 2001; Weitzman et al. 2015). The estimated prevalence of developmental delays and behaviour problems may substantially change depending on the instrument applied due to these psychometric considerations (de Albuquerque and da Cunha 2020).

Prevalence estimates of developmental delays and behavioural problems tend to be higher in low‐ and middle‐income countries like Brazil, where poverty, food insecurity, violence and combined biological and psychosocial risks are common. However, most Brazilian studies are regional, use small samples or employ different instruments, limiting comparability and hindering reliable national estimates. In the absence of representative national data, this study aimed to determine the overall prevalence rates in Brazilian children through a systematic review focused on addressing the following research question: ‘What is the prevalence of developmental delays and behavioural problems in Brazilian children assessed through screening tools?’

## 2 Methods

### 2.1 Search Strategy

We conducted a systematic review and meta‐analysis according to the new PRISMA statement (Page et al. 2021) and MOOSE guidelines (Stroup et al. 2000). The searches were carried out on Embase, Latin American and Caribbean Literature in Health Sciences (LILACS), PsycINFO, PubMed, Scopus and Web of Science databases and were finished on 25 September 2025. A non‐systematic search was also performed on Google Scholar and Open Grey, limited to the first 200 hits, to identify potentially eligible ongoing or unpublished studies. These sources were used to identify studies that could subsequently meet the eligibility criteria after publication in a peer‐reviewed journal. A manual search in the reference list of the studies included was also performed (see Supplementary Material S1 for full search syntax).

### 2.2 Inclusion Criteria

All published observational studies were included without any restrictions on publication year and language. Studies were included if:

- Prevalence of suspect development delays and behaviour problems was assessed through screening tools;
- They included Brazilian children in the age group 0 to 10 years old presenting not high risk (prematurity; low birth weight; hospitalised or other disorders or diagnosis of outcomes likely associated with developmental delays and behaviour problems).

### 2.3 Exclusion Criteria

Qualitative studies, reviews, letters, books, conference abstracts, studies carried out with animals, randomised clinical trials, theses and dissertations were excluded. Studies using diagnostic tests rather than screening instruments and instrument validation studies were also excluded. Studies with duplicated data from another included study were also excluded.

### 2.4 Study Selection

Study selection was performed at two stages. Stage 1—two independent authors assessed the titles/abstracts of references retrieved in the searches and exported to the Rayyan software. References whose titles/abstracts met the eligibility criteria were selected. References whose titles/abstracts did not provide enough information for an accurate decision on study inclusion or exclusion were assessed at Stage 2. Stage 2—full‐text assessment of references carried out by the same two independent authors; studies meeting the criteria were also selected. A third author would analyse the study in order to reach consensus if divergences on inclusion/exclusion between the two authors were observed.

### 2.5 Data Extraction

Data were extracted from included studies and transferred to an Excel spreadsheet by the two independent authors—divergences were solved by a third author. The extracted data were first author's last name, publication year, Brazilian state and region where the study was conducted, sample size, age of sampled subjects, screening tool used and data on development delay and/or behaviour disorder prevalence.

### 2.6 Risk of bias

Two blinded reviewers independently assessed the risk of bias based on Joanna Briggs Institute Critical Appraisal Checklist for Prevalence Studies. This tool comprises nine questions, called domains, which are used to assess the sample structure of the included study, that is, how the sample was selected and calculated, whether the study was described in details and how the recorded outcomes were assessed (Joanna Briggs Institute 2016). The answers could be ‘yes’, ‘unclear’, ‘no’ or ‘not applicable’. Decisions on scoring were agreed upon by all reviewers before the critical appraisal had started. Robvis (visualisation tool) was used to plot the figures.

A sensitivity analysis was performed to assess the robustness of the pooled prevalence estimate to the exclusion of studies classified as having a high risk of bias in the methodological quality assessment. The pooled prevalence estimate obtained in this analysis was compared with that of the primary meta‐analysis.

### 2.7 Meta‐Analysis

The prevalence of suspected developmental delays and behavioural problems identified through screening was expressed by means of relative or absolute frequencies and their 95% confidence intervals (95% CI). A proportion meta‐analysis was carried out to assess the overall pooled prevalence of developmental delays and behavioural problems. For each primary study, only the global/overall outcome classification reported was included in the meta‐analysis.

Some subgroups were subject to meta‐analysis aimed at assessing the pooled prevalence of suspected developmental delays and behaviour problems by taking into consideration the used instrument and the Brazilian geographic region.

The analysis was conducted in . Meta‐analysis of proportions was carried out by applying generalised linear mixed models with random effects, because a distribution of true effect sizes is expected to happen across studies. Statistical heterogeneity was quantified through the *I* ^2^ test, and heterogeneity was quantified through maximum likelihood as variance estimator. The meta‐analysis employed a random intercept logistic regression model, and the logit transformation was applied to the proportions.

## 3 Results

### 3.1 Study Selection

After removing duplicate records, 2369 out of the total of 3768 identified studies remained for title and abstracts screening. Subsequently, 249 studies were read for full‐text analysis at the second phase. Further information about the excluded studies and each exclusion criteria are available in Supplementary Material S2. Fifty‐nine (59) studies were selected for qualitative and quantitative syntheses at the end. An overview of the selection process is shown in Figure 1.

**FIGURE 1:**
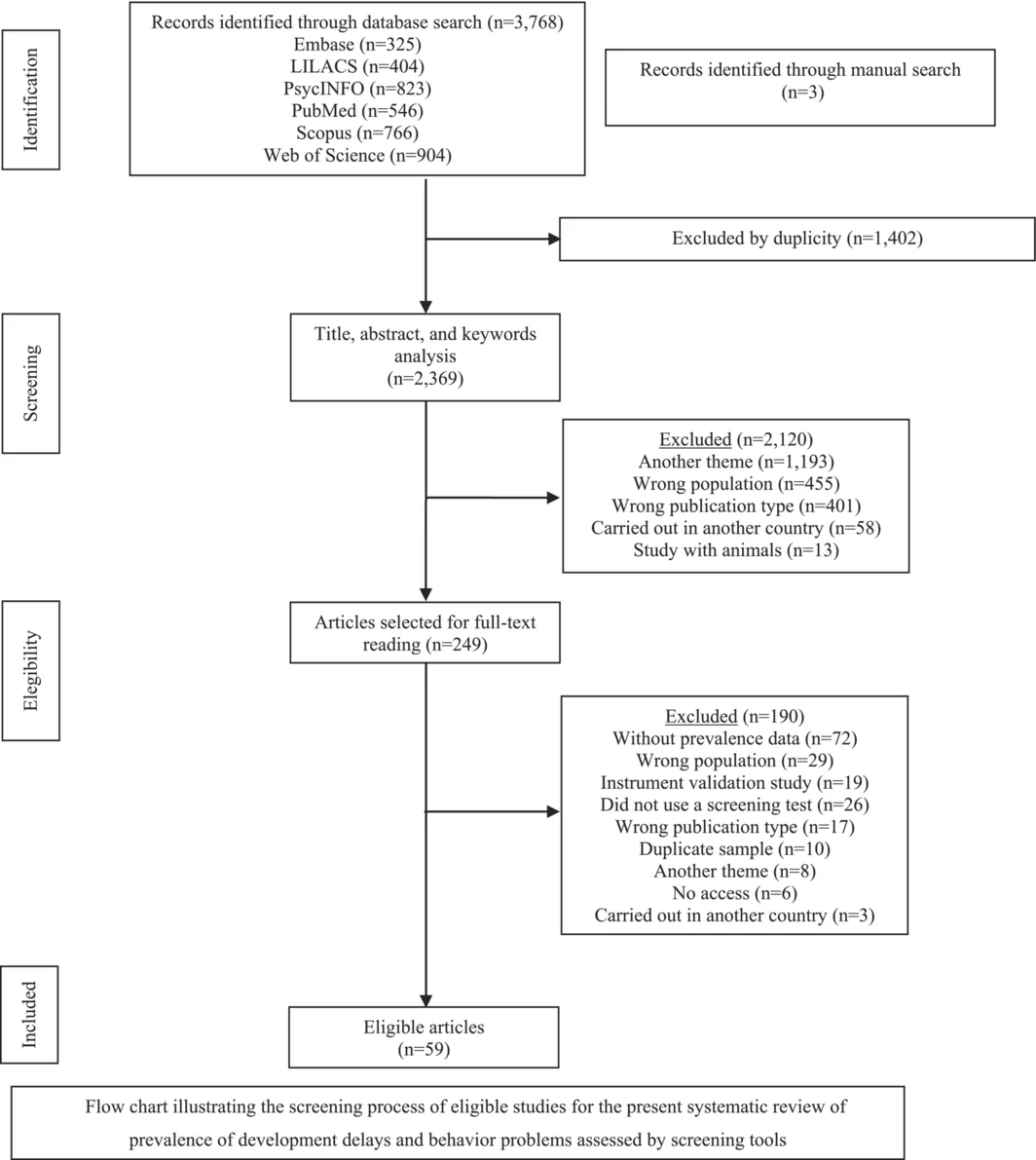
Flow chart illustrating the screening process of eligible studies for the present systematic review of prevalence of developmental delays and behaviour problems assessed by screening tools.

### 3.2 Characteristics of the Studies

The selected studies were conducted in 16 different states (59.3% of Brazilian states), mostly in the Southeastern region. Most of them were cross‐sectional and included children in the age group 0 to 2 years; sample size ranged from 30 (Souza et al. 2010) to 3907 (Moura et al. 2010) children. Child Behaviour Checklist and Denver Developmental Screening Test were the most often used screening instruments. Table 1 shows a summary of the evidence according to geographic region, screening instrument and outcome.

**TABLE 1:** Summary of the evidence according to geographic region, screening instrument and outcome (*n* = 59).

	Development, *n* (%)	Behaviour, *n* (%)	Both domains, *n* (%)	Total studies
Region				
North	2 (3.4)	1 (1.7)		3 (5.1)
Northeast	10 (17)	1 (1.7)	1 (1.7)	12 (20.4)
Midwest	1 (1.7)			1 (1.7)
Southeast	21 (35.6)	4 (6.8)	2 (3.4)	27 (45.8)
South	10 (17)	3 (5.1)		13 (22.1)
More than one	2 (3.4)			2 (3.4)
Not mentioned				1 (1.7)
Screening instrument				
ASQ‐3	4 (6.7)			4 (6.7)
BSDI	1 (1.7)			1 (1.7)
Bayley II or III	14 (23.7)			14 (23.7)
CBC		4 (6.7)		4 (6.7)
Denver II	19 (32.2)			19 (32.2)
Developmental Coordination Disorder Questionnaire	1 (1.7)			1 (1.7)
Developmental Surveillance Instrument	1 (1.7)		1 (1.7)	2 (3.4)
Gesell Early Screener	1 (1.7)			1 (1.7)
International Development and Early Learning Assessment			1 (1.7)	1 (1.7)
INTERGROWTH‐21st Neurodevelopment Assessment	1 (1.7)			1 (1.7)
Manual de Avaliação do Desenvolvimento Neuropsicomotor no Contexto da Atenção Integral das Doenças Prevalentes na Infância	1 (1.7)			1 (1.7)
Motor Development Scale	1 (1.7)			1 (1.7)
Movement Assessment Battery for Children	1 (1.7)			1 (1.7)
Peabody Developmental Motor Scale	1 (1.7)			1 (1.7)
SDQ		4 (6.8)	1 (1.7)	5 (8.5)
SWYC‐BR	2 (3.4)			2 (3.4)
Test of Gross Motor Development	1 (1.7)			1 (1.7)

Abbreviations: ASQ‐3, Ages and Stages Questionnaire Third Edition; Bayley‐II or III, Bayley Scales of Infant and Toddler Development, Second Edition or Third Edition; BSDI, Battelle Screening Developmental Inventory; CBC, Child Behaviour Checklist; Denver II, Denver Developmental Screening Test Second Edition; SDQ, Strengths and Difficulties Questionnaire; SWYC‐BR: Survey of Wellbeing of Young Children‐Brazilian Version.

Approximately 73% of the articles (*n* = 43) were published in the last 10 years. The prevalence of suspected developmental delays ranged from 0.68% (Coelho et al. 2024) to 77.74% (Guerreiro et al. 2016). The prevalence of suspected behavioural problems ranged from 1.35% (Coelho et al. 2024) to 52.45% (Altafim et al. 2018). All other relevant characteristics are presented in Table 2.

**TABLE 2:** Summary of the selected studies for a systematic review, which investigated the prevalence of suspect child developmental delays and behavioural problems accessed thought screening tools.

Study details	City, state and region	Study design	Population size/mean age or min/max	Screening instrument	Main findings
Altafim et al. (2018)	Ribeirão Preto, São Paulo, Southeast	Cross‐sectional	204/4.67 ± 1.46 years	SDQ	Behaviour problems scores: 52.5%
Amaro et al. (2015)	Diamantina, Minas Gerais, Southeast	Cross‐sectional	167/32.78 ± 11.35 months	Denver II	Suspect delay: 32.93%; abnormal delay: 5.99%; personal‐social: 15.57%; fine motor: 23.95%; language: 42.51%; gross motor: 13.17%
Anselmi et al. (2004)	Pelotas, Rio Grande do Sul, South	Prospective cohort	634/53 ± 3.6 months	CBC 4–18	Behaviour problems: 24% (clinical and borderline)
Araujo et al. (2020)	Campinas, São Paulo, Southeast	Cross‐sectional	132/8–10 months—69 (9.07 ± 0.68 months) with adequate environment and 63 (9.05 ± 0.72 months) with inadequate environment	Bayley‐III	Cognition: 0% below average (inadequate environment) and 3% below average (adequate environment); language: 6% below average (inadequate environment) and 1% below average (adequate environment); motor: 21% below average (inadequate environment) and 0% below average (adequate environment)
de Araújo and Oliveira‐Monteiro, (2020)	Santos, São Paulo, Southeast	Cross‐sectional	47/7 to 10 years	CBC 6–18	Behaviour problems: 19.14% (clinical and borderline)
Araujo et al. (2022)	Cruzeiro do Sul, Acre, North	Prospective cohort	735 children/12.7 ± 0.7 months) at 1 year and 819 children (23.8 ± 1.4 months) at 2 years	Denver II	General: 29.5% at 1 year and 51.0% at 2 years. Personal‐social: 14.8% at 1 year and 7.2% at 2 years. Fine motor: 9.3% at 1 year and 14% at 2 years. Gross motor: 5.9% at 1 year and 14.6% at 2 years. Language: 3.9% at 1 year and 37.1% at 2 years
Boo et al. (2018)	Fortaleza, Ceará, Northeast	Cross‐sectional	2755/18.7 ± 5.9 months	Denver II	Personal‐social: 23%; language: 20%; fine motor: 10% and gross motor: 10%
Brito et al. (2011)	Feira de Santana, Bahia, Northeast	Cross‐sectional	438/4 to 5 years	Denver II	General: 46.3%. Language: 50.26% at 4 years old and 41.93% at 5 years old. Fine motor: 22.05% at 4 years old and 39.43% at 5 years old. Gross motor: 10.77% at 4 years old and 10.04% at 5 years old. Personal‐social: 16.92% at 4 years old and 8.6% at 5 years old
Brito et al. (2022)	Parnaíba, Piauí, Northeast	Cross‐sectional	220/8.5 ± 6.1 months	Denver II	Suspect: 10.5%. Personal‐social: 2.3%; language: 6.8%, fine motor: 4.1%; gross motor: 3.6%
Camilo et al. (2024)	Maceió, Alagoas, Northeast	Cross‐sectional	224/28.8 ± 18.7 months	Denver II	General suspect delay: 63.8%
Caruzzo et al. (2020)	Maringá, Paraná, South	Cross‐sectional	357/3 to 5 years	Movement Assessment Battery for Children – 2	16.8% with low motor proficiency
Coelho et al. (2016)	Igrejinha, Rio Grande do Sul, South	Cross‐sectional	282/28 months	Denver II	General: 32% with suspect delay
Coelho et al. (2024)	São Paulo, São Paulo, Southeast	Cross‐sectional	443/69 ± 2.8 months	International Development and Early Learning Assessment	General: 0.7%. Motor: 1.4%; emergent literacy: 2.3%; emergent numeracy: 0.7%; socio‐emotional: 1.4%; executive function: 4.1%
Correia et al. (2019)	Fortaleza, Ceará, Northeast	Cross‐sectional	3566/31.8 ± 23.1 months	ASQ‐3 BR	At least one domain with development delay: 9.2%. Gross motor: 3.8%; communication: 2.0%; fine motor: 2.7%; problem‐solving: 2.8%; personal‐social 2.5%
Correia et al. (2023)	9 states at 4 regions	Cross‐sectional	108/8 ± 2.2 months	ASQ3‐BR	32.4% with atypical development
Cruz et al. (2021)	Pelotas, Rio Grande do Sul, South	Prospective cohort	3799/49.5 ± 1.7 months	CBC (1991)	Behaviour problems: 35.6%. Externalising: 44.4%. Internalising: 15.5%
Eickmann et al. (2008)	Recife, Pernambuco, Northeast	Cross‐sectional	76/4 to 24 months	Bayley‐II	Mental development: 30.3%; motor development: 13.2%
Ferreira et al. (2020)	Maringá, Paraná, South	Cross‐sectional	707/8.22 ± 1.17 years	Developmental Coordination Disorder Questionnaire ‐ Brazilian	Developmental coordination disorder: 31%
Filho et al. (2011)	São Luís, Maranhão, Northeast	Cross‐sectional	176/2 to 3 years	Gesell Early Screener	Developmental delay: 44.9%. Adaptive: 56.8%; gross motor: 0%; fine motor: 38.1%; language: 63.1%; personal‐social: 23.9%
Fukujima Goto et al. (2005)	Campinas, São Paulo, Southeast	Cross‐sectional	67/2 months	Bayley‐II	Mental: 29.85%; motor: 11.94%
Giacomini et al. (2024)	Cruzeiro do Sul, Acre, North	Prospective cohort	695/63.5 ± 2.71 months	SDQ	Total difficulties: 18.4%. Emotional problems: 22.6%; conduct problems: 24.8%; hyperactivity: 20.6%; peer problems: 21.9%; prosocial behaviour: 20%
Guerreiro et al. (2016)	Belém, Pará, North	Cross‐sectional	319/36 to 48 months	Denver II	General: 77.74% with suspect delay
Guimarães et al. (2013)	Belo Horizonte, Minas Gerais, Southeast	Cross‐sectional	291/2 to 24 months	Manual de Avaliação do Desenvolvimento Neuropsicomotor no Contexto da Atenção Integral das Doenças Prevalentes na Infância	Developmental delays: 31.7%; probable delay: 19.2%
Halpern et al. (2002)	Pelotas, Rio Grande do Sul, South	Prospective cohort	1363/12 months	Denver II	General: 34%; personal‐social: 5.5%; language: less than 1%; fine motor: 1.6%; gross motor: 15%
Lotto et al. (2025)	Ribeirão Preto, São Paulo, Southeast	Cross‐sectional	50/3.38 ± 0.92 months	SDQ	Behaviour problems: 26% (clinical and borderline)
Santa Maria‐Mengel and Linhares (2007)	Ribeirão Preto, São Paulo, Southeast	Cross‐sectional	120/22 months	Denver II	General suspect delay: 33%; language: 35%; fine motor: 19%; gross motor 21%
Martins et al. (2025)	Five cities in Southeast	Case–control	284/12 months	Bayley‐III	Cognitive: 2.8%; language: 16.3%; motor: 7.0%; delay in at least one domain: 20.4%
Melo et al. (2025)	Araranguá and Balneário Arroio do Silva, Santa Catarina, South	Cross‐sectional	498/41.17 ± 15.76 months	SWYC‐BR	Suspected alterations in overall development: 28.92%
Mezzari et al. (2019)	Porto Alegre, Rio Grande do Sul, South	Cross‐sectional	169/3.2 ± 1.7 years	Denver II	Language: 13.3%; gross motor: 6.7%; personal‐social: 33.3%
Mindell and Lee (2015)	NM	Cross‐sectional	1351/7.4 ± 2.89 months	ASQ‐3 BR	Communication: 5.5%; gross motor: 15.7%; fine motor: 13.8%; problem solving: 8.4%; personal‐social: 10.9%
Miquelote et al. (2012)	São Paulo, São Paulo, Southeast	Prospective cohort	32/9 ± 2.1 months and 15.2 ± 2 months	Bayley‐III	Motor: first assessment—low average: 25%; borderline: 6%. Second assessment—low average: 3%; extremely low: 3%. Cognitive—low average: 3%
Moraes et al. (2010)	São Paulo, São Paulo, Southeast	Cross‐sectional	35/0 to 6 years	Denver II	General: 28.6%; delay: 20%. Fine motor: 42%; personal‐social: 28.6%; language: 28.6%; gross motor: no evaluation
Morais et al. (2021)	Vale do Jequitinhonha, Minas Gerais, Southeast	Cross‐sectional	147/30.05 ± 4.17 months	Bayley‐III	Cognitive scale: low average (18.4%); Boderline (2.7%). Motor scale: low average (10.9%); borderline (0%)
Moura et al. (2010)	Pelotas, Rio Grande do Sul, South	Prospective cohort	3907 at 12 months and 3782 at 24 months	BSDI	12 months: 9.98%. 24 months: 3.20%
Negrão et al. (2020)	Ribeirão Preto, São Paulo, Southeast	Prospective cohort	1006/13 to 30 months	Bayley‐III	Fine motor: 8.8%; gross motor: 9.9%; cognitive: 14.4%
Neves et al. (2016)	Diamantina, Minas Gerais, Southeast	Cross‐sectional	92/24 to 36 months	Bayley‐III	Language: 28.3%; cognitive: 28.6%
Neves et al. (2020)	Pelotas, Rio Grande do Sul, South	Prospective cohort	3776/24 months	INTERGROWTH‐21st Neurodevelopment Assessment	Global: 10.2%; language: 10%; cognitive: 11.4%; motor: 10%
Nobre et al. (2021)	NM, Minas Gerais, Southeast	Cross‐sectional	180/35 ± 4.5 months	Bayley‐III	Cognitive: 45.19%; language: 30.92%; gross motor: 72.41%; fine motor: 31.40%
Oliveira et al. (2021)	Brasília, Distrito Federal, Midwest	Cross‐sectional	1004/ <2 years	Denver II	Risk of developmental delay: 15.1%.
Oliveira, Palombo, et al. (2023)	Itupeva, São Paulo, Southeast	Cross‐sectional	348/27.4 months	Developmental Surveillance Instrument	Motor developmental delay: 27.6%; socioemotional developmental delay: 17.2%
Pinheiro et al. (2024)	5 Southeast Brazilian cities	Prospective cohort	449/6 and 12 months	SWYC‐BR	Suspect developmental delay at 6 months: 29.5%; and 24.7% at 12 months
Ramos and Della Barba (2021)	Itatiba, Sao Paulo, Southeast	Cross‐sectional	392/5 a 50 months	ASQ3‐BR	Communication: 12.18%; gross motor: 11.93%; fine motor: 19.03%; problem‐solving: 13.96%; personal‐social: 22.08%
Ré et al. (2018)	São Paulo, São Paulo, Southeast	Cross‐sectional	424/5 to 10 years	Test of Gross Motor Development‐2nd edition	Very low motor competence: 39.4%; low motor competence: 19.1%
Reyes et al. (2019)	Pelotas, Rio Grande do Sul, South	Cross‐sectional	596/7–8 years	SDQ	Behaviour problems: 30.7%
Rezende et al. (2003)	São Paulo, São Paulo, Southeast	Prospective cohort	36 at 1^st^ evaluation e 33 at 2^nd^ evaluation	Denver II	Language—first evaluation: 15.1%; second evaluation: 0%. Personal‐social—first evaluation: 19.4%; second evaluation: 0%
Ribeiro et al. (2014)	São Paulo, Southeast	Cross‐sectional	65/11 to 12 months	Denver II	Global developmental delay: 43.1%; language: 40%; fine motor: 20.0%; personal‐social: 44.6%; gross motor: 0%
Santos et al. (2009)	Piracicaba, São Paulo, Southeast	Cross‐sectional	145/6 to 38 months	Peabody Developmental Motor Scale‐2	Global: 17%; reflexes: 13.8%; stationary skills: 3.4%; locomotion skills: 16%; object manipulation: 9%
Santos et al. (2016)	Salvador, Bahia, Northeast	Cross‐sectional	349/49 to 72 months	CBC (1991)	Behaviour problems: 23.5%. Internalising problems: 9.7%; Externalising problems: 25.2%
dos Santos et al. (2025)	Nazaré and Aratuípe, Bahia, Northeast	Cross‐sectional	66/12 months	Bayley‐III	Cognitive: 4.8%; language: 4.8%; motor: 1.6%; adaptive behaviour: 14.3%
Schiavo and Perosa (2020)	3 inland cities of São Paulo, Southeast	Prospective cohort	139/6 and 14 months	Denver II	6 months: global: 40%; personal‐social: 13%; adaptative motor: 19%; language: 4%; broad motor: 30% 14 months: global: 31%; personal‐social: 9%; adaptative motor: 3%; language: 23%; broad motor: 15%
Severiano et al. (2017)	Santa Cruz, Rio Grande do Norte, Northeast	Cross‐sectional	99/14.57 months	Developmental Surveillance Instrument	At least one motor milestone absent: 29.29%
Silva et al. (2015)	João Pessoa, Paraíba, Northeast	Cross‐sectional	112/14.4 ± 3 months	Denver II	Suspect: 23.2%; delay: 29.5%; personal‐social: 17%; fine motor: 16.1%; language: 19.6%; gross motor: 17.0%
Silva et al. (2017)	Matinhos, Paraná, South	Cross‐sectional	91/8 to 10 years	Motor Development Scale	Risk of developmental delay: 59.3%
Sousa et al. (2022)	Picos, Piauí, Northeast	Cross‐sectional	287/1 to 24 months	SWYC‐BR	Suspect developmental delay: 12.7%. Suspect of socioemotional development: 42.2%
Souza et al. (2010)	Piracicaba, São Paulo, Southeast	Prospective cohort	30/12 ± 0.61 months at 1^st^ evaluation and 17 ± 0.33 months at 2^nd^ evaluation	Bayley‐III	First evaluation, suspected delays: 26.1%; second evaluation:16.7%. 13% had suspected delays in overall performance.
Tella et al. (2018)	São Paulo, São Paulo, Southeast	Cross‐sectional	444/6 to 9 months	Bayley‐III	Motor: 10.6%; language: 7.46%; cognitive: 1.92%
Veleda et al. (2011)	Rio Grande, Rio Grande do Sul, South	Cross‐sectional	220/8 to 12 months	Denver II	Suspected delay in neuropsychomotor development: 20.5%
Wehby and Trujillo (2017)	7 cities, 6 different regions	Cross‐sectional	488/3 to 24 months	Bayley‐III	At high risk of developing neurodevelopmental problems: 21%
Zaqueu et al. (2015)	Barueri, São Paulo, Southeast	Cross‐sectional	92/21.78 ± 1.97 months	Denver II	Neuropsychomotor development delays: 28.3%

ASQ‐3: Ages and Stages Questionnaire Third Edition; BSDI: Battelle Screening Developmental Inventory; Bayley‐II: Bayley Scales of Infant and Toddler Development, Second Edition; Bayley‐III: Bayley Scales of Infant and Toddler Development, Third Edition; CBC: Child Behaviour Checklist; Denver II: Denver Developmental Screening Test Second Edition; NM: Not mentioned; SDQ: Strengths and Difficulties Questionnaire; SWYC‐BR: Survey of Wellbeing of Young Children‐Brazilian Version.

### 3.3 Study Methodological Quality Assessment

In total, 26 (44.1%) of the 59 included studies recorded low risk of bias based on JBI criteria. Domains presenting the highest rate of uncertain judgements regarded standardised measurements applicable to outcomes (*n* = 14; 23.7%) and study participant appropriate selection (*n* = 8; 13.6%). Domains accounting for the highest rate of unfavourable judgements regarded adequate sample size calculation (*n* = 44; 74.6%) and the validity of adopted methods (*n* = 28; 47.4%; see Supplementary Material S3).

### 3.4 Meta‐Analysis

The estimated pooled prevalence of suspected developmental delays across the articles included in the overall meta‐analysis was 28.72% (95% CI: 23.21%–34.95%), whereas the pooled prevalence of behaviour problems was 28.17% (95% CI: 22.05%–35.23%).

The sensitivity analysis for suspected developmental delays excluded five studies classified as having a high risk of bias. After their exclusion, the pooled prevalence was 28.0%, which was similar to the estimate obtained in the primary meta‐analysis including these studies. This finding indicates that the pooled prevalence estimate was not substantially affected by the inclusion of studies classified as having a high risk of bias (Supplementary Material S4). For behavioural problems, a sensitivity analysis based on risk of bias was not performed because none of the studies included in the meta‐analysis were classified as having a high risk of bias.

With respect to the country's geographic region, the prevalence of suspected developmental delays ranged from 15.14% in the Midwestern region to 54.65% in the Northern region. The prevalence of suspected behavioural problems ranged from 18.42% in the Northern region to 32.10% in the Northeastern region (Figures 2 and 3).

**FIGURE 2:**
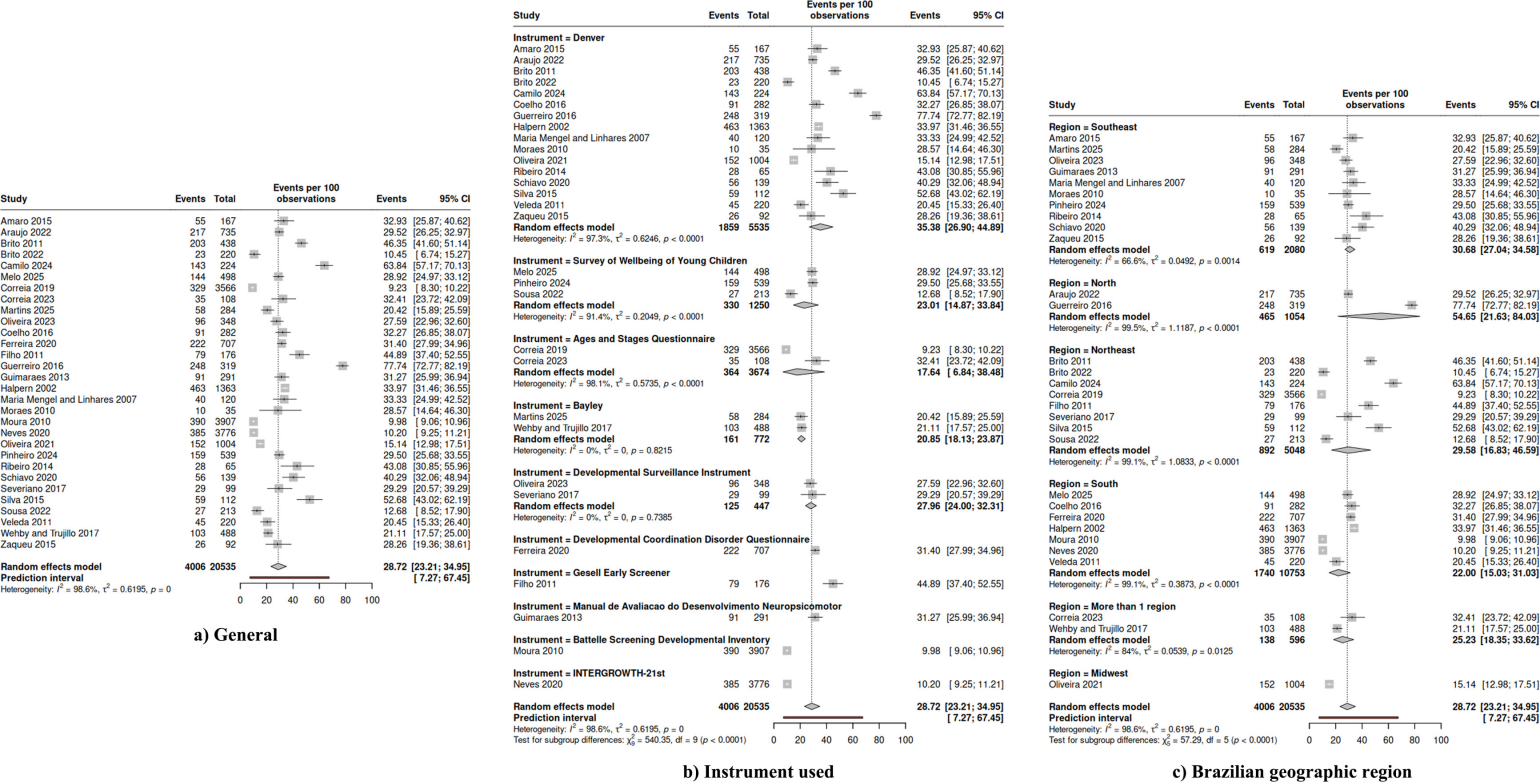
Forest plot of prevalence of suspected developmental delays among Brazilian children as assessed by screening tools. R Studio (Posit PBC 2024).

**FIGURE 3:**
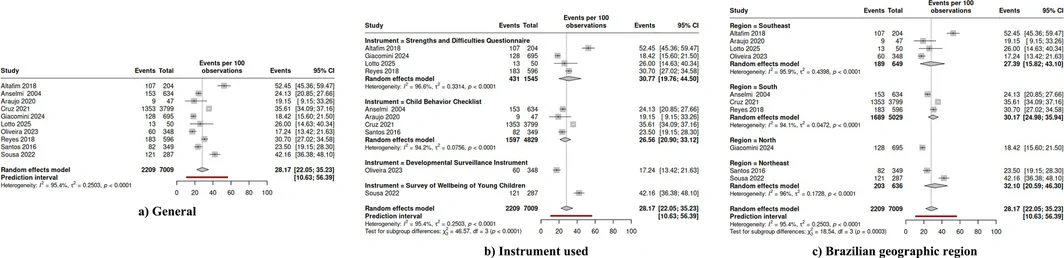
Forest plot of prevalence of suspected behavioural problems among Brazilian children as assessed by screening tools. R Studio (Posit PBC, 2024).

The prevalence of suspected developmental delay ranged from 9.98%, based on the Batelle Screening Developmental Inventory, to 44.89%, based on the Gesell Early Screener. The prevalence of suspected behavioural problems ranged from 17.24% in the Developmental Surveillance Instrument to 42.16% in the Survey of Wellbeing of Young Children (Figures 2 and 3).

The subgroup meta‐analyses also allowed observing significant differences in the prevalence of outcomes (development and behaviour) when compared the instruments used and the geographic region (*p* < 0.01; Figures 2 and 3).

## 4 Discussion

This systematic review synthesised the available evidence on prevalence of suspected developmental delays and behavioural problems in Brazilian children assessed through screening tools. The meta‐analysis estimated a pooled prevalence of approximately 28% for suspected developmental delays and 28% for behavioural problems across the populations represented in the included studies. However, these estimates should be interpreted with caution, given the substantial clinical, methodological, geographic and measurement heterogeneity across studies, including differences in age groups, sampling strategies, geographic coverage, screening instruments and outcome definitions. Nevertheless, the findings indicate that suspected developmental and behavioural problems are frequently identified in the populations assessed and underscore the potential value of developmental and behavioural screening as part of child health surveillance, particularly when followed by appropriate clinical assessment and diagnostic evaluation when indicated.

Our study complies with current US estimates, according to which, more than one in five children can be expected to fail screening tests (Glascoe 2015). Some factors can have negative influence on the poor performance of screening tests, such as families facing psychosocial risks, housing instability, parental depression, chemical dependence, violence, limited parental schooling and economic disadvantages (Glascoe 2015; de Souza Morais et al. 2021; Weitzman et al. 2015).

Moreover, the present meta‐analysis identified a wide range in prevalence estimates across primary studies, and this finding meets a systematic review (de Lima et al. 2016) that included publications from 2005 to 2015 and found suspected developmental delays rates ranging from 0% to 46.3% in Brazilian children without risk factors. These differences can be mostly attributed to lack of uniformity in assessments applied to the used instruments (Rocha et al. 2013), which hinders direct comparisons between them.

We also found that instruments developed in other countries were predominantly used, including the Denver Developmental Screening Test, whose cutoff points, even after validation, are often those of the original or adapted versions (Amaro et al. 2015; Boo et al. 2018; Coelho et al. 2016; de Lima et al. 2016; Schiavo and Perosa 2020); however, they can vary across regions due to cultural and socioeconomic differences. Identification rates exceed 70% when health professionals use screening instruments with adequate psychometric properties. On the other hand, only 30%–40% of at‐risk children are detected when informal checklists or tests with poor measurement properties are adopted (Glascoe 2015).

Systematic reviews (Bliznashka et al. 2025; Rocha et al. 2013) showed that the use of screening tests developed in high‐income countries, without adequate translation and cross‐cultural adaptation for low‐ and middle‐income countries, such as Brazil, can compromise the assessment of at‐risk children. It is so because these instruments do not present standardised performance for the assessed population and have manuals that have not yet been translated, which further hinders their proper application and potentially leads to negative consequences for child health (Bliznashka et al. 2025; Rocha et al. 2013). Moreover, few studies synthesised the evidence available on the psychometric properties of developmental and behavioural screening instruments (Bliznashka et al. 2025). The use of standardised instruments in research and routine clinical practice increases reliability and rigour in identifying delays, besides supporting the monitoring of at‐risk children. The instruments used should be sensitive enough to detect early signs of delay in children from different regions and socioeconomic backgrounds and allow appropriate guidance and early interventions.

The current review also identified that the studies were only conducted in approximately 60% of Brazilian states, although it covered all regions, and this finding points out the need for assessing developmental and behavioural screening in other states. Furthermore, the meta‐analysis identified the Northern region as the most prevalent one for suspected developmental delays and the Northeastern region as the most prevalent region for suspected behavioural problems. However, these regional differences should be interpreted cautiously, as they may also reflect differences in sampling strategies, screening instruments, age distributions, study settings, recruitment strategies and methodological quality rather than true regional differences in child development or behaviour.

Some factors may potentially contribute to the observed differences between regions. Firstly, one finds the social and regional inequalities historically featuring Brazil (Guerreiro et al. 2016). Some of these region's features include low socioeconomic status and vulnerabilities of these children's family or community environment, which are among the main risk factors for developmental delays and behavioural problems (Araujo et al. 2022; Guerreiro et al. 2016; Santos et al. 2016). Other factors have also emerged as aggravating, including mother–child interaction impairments, low parental schooling, mental illness and substance abuse (such as alcohol and/or drugs) (Araujo et al. 2022; Santos et al. 2016; Sousa et al. 2022). Nevertheless, these factors were not directly assessed as determinants of the regional differences in the present meta‐analysis, and therefore, these findings should not be interpreted as evidence of a causal relationship between socioeconomic vulnerability and the observed regional estimates.

It is important to emphasise the importance of strengthening child health surveillance and developmental and behavioural monitoring within primary care, while recognising that the pooled prevalence estimates alone do not establish the effectiveness, cost‐effectiveness or potential harms of expanding universal screening across different regions in the country. In this context, the findings underscore the need for nationally representative epidemiological studies and for continued strengthening of existing child health monitoring strategies within the Brazilian public healthcare system. The Brazilian *Child Health Handbook* represents an important tool in this regard, which currently includes a section to be completed by health professionals about the continuous monitoring of developmental milestones from birth to the age of six years.

It is essential for health professionals to get proper training and to acquire the necessary skills to administer and interpret the instruments, even after these actions are taken, in order to enable early interventions. It is also crucial for professionals to select instruments that have been culturally adapted and validated for the target population (de Albuquerque and da Cunha 2020).

### 4.1 Limitations and Strengths

This review has some important limitations that should be mentioned. Firstly, there was substantial clinical, methodological, geographic and measurement heterogeneity among the included studies. The studies differed in children's age groups, sampling strategies, sample sizes, geographic settings, screening instruments and definitions and thresholds used to identify suspected developmental delays and behavioural problems. In particular, the use of different screening instruments may have contributed substantially to the variability in prevalence estimates, as these instruments may assess different domains, use different scoring systems and apply different thresholds for classification. Secondly, methodological quality varied across the included studies as most of the included studies did not provide detailed information on sample size calculations. In studies where the population is selected by the researchers, prevalence estimates tend to be higher compared with those obtained from randomly selected samples (Oliveira, Pauletto, et al. 2023). However, even after excluding studies with high risk of bias in the sensitivity analysis, the pooled prevalence is similar to that of the primary analysis, suggesting that the overall estimate for this outcome was not substantially affected by these studies.

Thirdly, the geographic distribution of the evidence was uneven, with limited evidence from some regions. Therefore, regional differences in pooled prevalence should be interpreted cautiously, particularly for the North, which was represented by only two studies. Rather than establishing regional differences, the findings highlight the scarcity and uneven distribution of epidemiological evidence across Brazil. Although subgroup analyses and heterogeneity statistics helped explore variability, they cannot overcome differences in study design, populations, sampling strategies and measurement approaches.

The diversity of instruments used and the high heterogeneity among the selected studies were key factors influencing its construction. Screening instruments may differ in their sensitivity, specificity, cutoff points, cultural adaptation, validation procedures, informant, administration method and age‐specific norms, and these differences may contribute to variability in the observed prevalence. Consequently, the pooled estimates should be interpreted cautiously, particularly considering the methodological variability and the absence of nationally representative population‐based studies.

Furthermore, our decision to exclude unpublished studies can contribute to availability or publication bias and may affect the representativeness of the available evidence.

Several strengths of the present review should be noted. Previous systematic reviews have been limited in scope, because some of them assessed only a single screening instrument (de Lima et al. 2016), whereas others investigated only one developmental outcome (Bliznashka et al. 2025; de Albuquerque and da Cunha 2020). Additionally, some studies relied only on a limited number of databases (de Albuquerque and da Cunha 2020) or on a limited search period (Bliznashka et al. 2025). On the other hand, this review imposed no limits on developmental or behavioural domains and included both multi‐domain and general screening tools, besides being conducted by a rigorously trained research team.

Furthermore, when it comes to different instruments used for developmental and behavioural screening, this systematic review provides a comprehensive overview of the available evidence and highlights important gaps in the Brazilian literature. The substantial variability observed across instruments also underscores the importance of using culturally adapted and appropriately validated screening tools, as well as of ensuring that trained professionals conduct screening tests' administration and readministration in clinical practices.

## 5 Conclusion

The available evidence indicates that suspected developmental delays and behavioural problems are frequently identified through screening tools among the populations represented in Brazilian studies, with pooled prevalence estimates of 28.72% and 28.17%, respectively. However, these estimates should not be interpreted as nationally representative prevalence estimates, given the substantial clinical, methodological, geographic and measurement heterogeneity across studies and the uneven distribution of evidence across Brazilian regions. The significant differences observed according to geographic region and screening instrument further highlight the importance of considering contextual and measurement‐related factors when interpreting pooled estimates.

Additional studies are recommended to assess the impact of different settings, including the use of representative and randomised samples, for the application of screening instruments. Future studies should adopt representative and randomised samples; focus on translating, validating and establishing strong psychometric properties, including reliability, validity, sensitivity and specificity for both imported and locally developed tools; and promote the development of fast, simple and accessible screening instruments for routine use across healthcare settings.

## Author Contributions

**Rafaela Cristina Vieira e Souza:** writing – review and editing, writing – original draft, investigation, formal analysis, data curation, conceptualization. **Larissa Bueno Ferreira:** writing – review and editing, formal analysis, data curation. **Tamires Nunes dos Santos:** writing – review and editing, formal analysis, data curation. **Luana Caroline dos Santos:** funding acquisition, writing – review and editing, supervision.

## Funding

This study was supported by the Fundação de Amparo a Pesquisa do Estado de Minas Gerais (under grant number APQ‐01782‐10) and Conselho Nacional de Desenvolvimento Científico e Tecnológico (productivity scholarship under grant number 301555/2019‐2). The funders had no role in study design, methods, decision to publish or preparation of the manuscript.

## Conflict of Interests

The authors declare no conflicts of interest.

## Supporting information


**SUPPLEMENTARY MATERIAL S1** Search conducted on 25 September 2025.

**SUPPLEMENTARY MATERIAL S2** Articles excluded and the reasons for exclusion (*n* = 190).

**SUPPLEMENTARY MATERIAL S3** Risk of bias assessment (ROBVIS visualisation tool, 2025).

**SUPPLEMENTARY MATERIAL S4** Sensitivity analysis of prevalence of suspected developmental delays among Brazilian children as assessed by screening tools.

**Data S1:** Supporting Information.

## Data Availability

All relevant data are available within the paper and supporting information.
