# Supplementary material for: Prevalence of Suspected Developmental Delays and Behavioural Problems Identified Using Screening Tools Among Brazilian Children: A Systematic Review and Meta‐Analysis
Source: Child Care Health Dev. 2026 Nov 14;52(6):e70379. doi: 10.1111/cch.70379 (PMC13768938; doi:10.1111/cch.70379)
Supplement: Supplementary file 1 [file CCH-52-e70379-s001.docx]

Supplementary material 1. Search conducted on September 25, 2025

**Pubmed** n=546

((("Child Development"[Mesh] OR "Development, Child" OR "Infant Development" OR "Development, Infant" OR "Child Behavior"[Mesh] OR "Behavior, Child" OR "Infant Behavior" OR "Developmental Delay" OR "Problem Behavior"[Mesh] OR "Behavior, Problem" OR "Behaviors, Problem" OR "Problem Behaviors" OR "Behavioral Problem" OR "Behavioral Problems" OR "Problem, Behavioral" OR "Problems, Behavioral" OR "Disruptive Behavior" OR "Behavior, Disruptive" OR "Behaviors, Disruptive" OR "Disruptive Behaviors" OR "Dysfunctional Behavior, Psychology" OR "Behavior, Psychology Dysfunctional" OR "Behaviors, Psychology Dysfunctional" OR "Dysfunctional Behaviors, Psychology" OR "Psychology Dysfunctional Behavior" OR "Psychology Dysfunctional Behaviors")) AND (("Screening Tool" OR Screening OR Scale OR Inventory OR Tool OR Instruments))) AND (("Brazil"[Mesh] OR Brazilian))

**Embase** n=325

('child development' OR 'infant development' OR 'child behavior' OR 'infant behavior' OR 'developmental delay' OR 'behavioral problems' OR 'behavior problems') AND ('screening tool' OR screening OR scale OR inventory OR tool OR instruments) AND (brazil OR brazilian)

**Lilacs** n=404

("Desenvolvimento Infantil" OR "Desenvolvimento da Criança" OR "Desenvolvimento da Criança em Idade Pré-Escolar" OR "Desenvolvimento da Criança Pré-Escolar" OR "Desenvolvimento das Crianças" OR "Desenvolvimento das Crianças em Idade Pré-Escolar" OR "Desenvolvimento de Criança" OR "Desenvolvimento de Crianças" OR "Desenvolvimento de Lactentes" OR "Desenvolvimento de Pré-Escolares" OR "Desenvolvimento do Lactente" OR "Desenvolvimento do Pré-Escolar" OR "Desenvolvimento dos Lactentes" OR "Desenvolvimento dos Pré-Escolares" OR "Desenvolvimento Pré-Escolar" OR "Child Development" OR "Development, Child" OR "Development, Infant" OR "Infant Development" OR "Desarrollo Infantil" OR "Desarrollo de Lactantes" OR "Desarrollo de los Lactantes" OR "Desarrollo de los Niños" OR "Desarrollo de los Niños en Edad Preescolar" OR "Desarrollo de los Preescolares" OR "Desarrollo de Niños" OR "Desarrollo de Preescolares" OR "Desarrollo del Lactante" OR "Desarrollo del Niño" OR "Desarrollo del Niño en Edad Preescolar" OR "Desarrollo del Preescolar" OR "Desarrollo Preescolar" OR "Deficiências do Desenvolvimento" OR "Desvios do Desenvolvimento Infantil" OR "Transtornos de Atraso do Desenvolvimento" OR "Transtornos do Desenvolvimento Infantil" OR "Transtornos Específicos do Desenvolvimento Infantil" OR "Developmental Disabilities" OR "Child Development Deviation" OR "Child Development Deviations" OR "Child Development Disorder" OR "Child Development Disorders" OR "Child Development Disorders, Specific" OR "Development Deviation, Child" OR "Development Deviations, Child" OR "Development Disorder, Child" OR "Development Disorders, Child" OR "Developmental Delay Disorder" OR "Developmental Delay Disorders" OR "Developmental Disability" OR "Deviation, Child Development" OR "Disabilities, Developmental" OR "Disability, Developmental" OR "Discapacidades del Desarrollo" OR "Desviaciones del Desarrollo Infantil" OR "Trastornos de Retraso en el Desarrollo" OR "Trastornos del Desarrollo Infantil" OR "Trastornos Específicos del Desarrollo Infantil" OR "Comportamento Infantil" OR "Comportamento da Criança" OR "Comportamentos das Crianças" OR "Comportamentos Infantis" OR "Child Behavior" OR "Behavior, Child" OR "Conducta Infantil" OR "Comportamiento de la Niñez" OR "Comportamiento del Niño" OR "Comportamiento en la Niñez" OR "Comportamiento Infantil" OR "Comportamientos de la Niñez" OR "Comportamientos de los Niños" OR "Comportamientos Infantiles" OR "Conducta de la Niñez" OR "Conducta del Niño" OR "Conducta en la Niñez" OR "Conductas de la Niñez" OR "Conductas de los Niños" OR "Conductas en la Niñez" OR "Conductas Infantiles" OR "Transtornos do Comportamento Infantil" OR "Distúrbios do Comportamento em Crianças" OR "Transtornos da Conduta em Crianças" OR "Transtornos do Comportamento da Criança" OR "Transtornos do Comportamento em Crianças" OR "Child Behavior Disorders" OR "Behavior Disorder, Child" OR "Behavior Disorder, Childhood" OR "Behavior Disorders, Child" OR "Child Behavior Disorder" OR "Childhood Behavior Disorder" OR "Childhood Behavior Disorders" OR "Disorder, Childhood Behavior" OR "Disorders, Child Behavior" OR "Trastornos de la Conducta Infantil" OR "Trastornos de Comportamiento Infantil" OR "Trastornos de Conducta del Niño" OR "Trastornos de Conducta en la Niñez" OR "Trastornos de Conducta en Niños" OR "Trastornos de Conducta Infantil" OR "Trastornos de la Conducta del Niño" OR "Trastornos de la Conducta en la Infancia" OR "Trastornos del Comportamiento de la Infancia" OR "Trastornos del Comportamiento de la Niñez" OR "Trastornos del Comportamiento del Niño" OR "Trastornos del Comportamiento en la Infancia" OR "Trastornos del Comportamiento en la Niñez") AND ("Programas de Rastreamento" OR "Exame Coletivo" OR "Identificação Sistemática" OR "Rastreamento" OR "Screening" OR "Triagem de Massa" OR "Mass Screening" OR "Mass Screenings" OR "Screening, Mass" OR Screenings OR "Screenings, Mass" OR "Tamizaje Masivo" OR Cribado OR "Cribado Masivo" OR "Cribado Sistemático" OR "Detección Selectiva" OR "Detección Sistemática" OR "Examen Colectivo" OR "Identificación Sistemática" OR "Screening Masivo" OR Tamización OR "Tamización Masiva" OR Tamizaje) AND ("Brasil" OR "Brazil")

**PsycINFO** n=823

("Child Development" OR "Infant Development" OR "Child Behavior" OR "Infant Behavior" OR "Developmental Delay" OR "Behavioral Problems" OR "Behavior Problems") AND ("Screening Tool" OR Screening OR Scale OR Inventory OR Tool OR Instruments) AND (Brazil OR Brazilian)

**Scopus** n=766

("Child Development" OR "Infant Development" OR "Child Behavior" OR "Infant Behavior" OR "Developmental Delay" OR "Behavioral Problems" OR "Behavior Problems") AND ("Screening Tool" OR Screening OR Scale OR Inventory OR Tool OR Instruments) AND (Brazil OR Brazilian)

**Web of science** n=904

("Child Development" OR "Infant Development" OR "Child Behavior" OR "Infant Behavior" OR "Developmental Delay" OR "Behavioral Problems" OR "Behavior Problems") AND ("Screening Tool" OR Screening OR Scale OR Inventory OR Tool OR Instruments) AND (Brazil OR Brazilian)
