# Supplementary material for: Prevalence of Suspected Developmental Delays and Behavioural Problems Identified Using Screening Tools Among Brazilian Children: A Systematic Review and Meta‐Analysis
Source: Child Care Health Dev. 2026 Nov 14;52(6):e70379. doi: 10.1111/cch.70379 (PMC13768938; doi:10.1111/cch.70379)
Supplement: Supplementary file 2 [file CCH-52-e70379-s002.docx]

Supplementary material 3. Risk of bias assessment (ROBVIS visualization tool, 2025)
