# Supplementary material for: Prevalence of Suspected Developmental Delays and Behavioural Problems Identified Using Screening Tools Among Brazilian Children: A Systematic Review and Meta‐Analysis
Source: Child Care Health Dev. 2026 Nov 14;52(6):e70379. doi: 10.1111/cch.70379 (PMC13768938; doi:10.1111/cch.70379)
Supplement: Supplementary file 3 [file CCH-52-e70379-s003.docx]

**Supplementary material 2.** Articles excluded and the reasons for exclusion (n=190)

| **References** |  | **Author** | **Reasons for exclusion*** |
| --- | --- | --- | --- |
| [1] |  | Almeida et al., 2008 | 3 |
| [2] |  | Alvarenga et al., 2016 | 2 |
| [3] |  | Andrade et al., 2005 | 7 |
| [4] |  | Anunciação et al., 2019 | 3 |
| [5] |  | Ávila et al., 2025 | 1 |
| [6] |  | Bandeira et al., 2006 | 4 |
| [7] |  | Barros et al., 2010 | 1 |
| [8] |  | Batista et al., 2023 | 1 |
| [9] |  | Bertoldi et al., 2020 | 1 |
| [10] |  | Beteli, 2006 | 5 |
| [11] |  | Bobbio et al., 2007 | 1 |
| [12] |  | Bobbio et al., 2010 | 1 |
| [13] |  | Bolsoni-Silva and Loureiro, 2023 | 2 |
| [14] |  | Bolsoni-Silva et al., 2010 | 1 |
| [15] |  | Bolsoni-Silva et al., 2011 | 7 |
| [16] |  | Borsa et al., 2011 | 5 |
| [17] |  | Brentani et al., 2016 | 1 |
| [18] |  | Brito and Onis, 2004 | 1 |
| [19] |  | Brunstein and Tiellet Nunes, 2003 | 9 |
| [20] |  | Caçola et al., 2011 | 3 |
| [21] |  | Caetano et al., 2016 | 5 |
| [22] |  | Camargo-Figuera et al., 2014 | 4 |
| [23] |  | Camargos et al., 2016 | 2 |
| [24] |  | Campos et al., 2012 | 1 |
| [25] |  | Carvalho da Silva et al., 1993 | 8 |
| [26] |  | Castelli et al., 2015 | 1 |
| [27] |  | Castro et al., 2025 | 1 |
| [28] |  | Castro et al., 2025 | 1 |
| [29] |  | Cavaggioni et al. 2020 | 2 |
| [30] |  | Christovão et al., 2024 | 2 |
| [31] |  | Coelho et al., 2021 | 1 |
| [32] |  | Costa et al., 2021 | 7 |
| [33] |  | Cunha and Melo, 2005 | 5 |
| [34] |  | Da Costa et al., 2021 | 1 |
| [35] |  | Da Fonseca Filho et al., 2021 | 2 |
| [36] |  | Da Silva et al., 2019 | 3 |
| [37] |  | Da Silva et al., 2023 | 1 |
| [38] |  | Da Silva et al., 2023 | 1 |
| [39] |  | De Almeida Filho, 1982 | 8 |
| [40] |  | De Andrade Leão et al., 2024 | 1 |
| [41] |  | De Borba et al., 2015 | 1 |
| [42] |  | De Mello et al., 2014 | 4 |
| [43] |  | De Oliveira et al., 2017 | 1 |
| [44] |  | De Oliveira et al., 2021 | 1 |
| [45] |  | De Oliveira et al., 2025 | 2 |
| [46] |  | Defilipo et al., 2012 | 7 |
| [47] |  | Defilipo et al., 2021 | 4 |
| [48] |  | Delgado et al., 2020 | 2 |
| [49] |  | Dias et al., 2013 | 1 |
| [50] |  | Domingues et al., 2014 | 1 |
| [51] |  | Dornelas et al., 2016 | 2 |
| [52] |  | Dos Santos and Valentini, 2020 | 5 |
| [53] |  | Dos Santos et a., 2020 | 4 |
| [54] |  | Dos Santos et al., 2022 | 4 |
| [55] |  | Drachler et al., 2007 | 3 |
| [56] |  | Duarte et al., 2022 | 1 |
| [57] |  | Dumcke et al., 2020 | 2 |
| [58] |  | Eickmann et al., 2003 | 1 |
| [59] |  | Eickmann et al., 2007 | 1 |
| [60] |  | Emerich et al., 2012 | 2 |
| [61] |  | Fattore et al., 2017 | 2 |
| [62] |  | Fattore et al., 2017 | 1 |
| [63] |  | Fernandes et al., 2012 | 2 |
| [64] |  | Fernandes et al., 2020 | 7 |
| [65] |  | Fernandes et al., 2022 | 3 |
| [66] |  | Fernandes et al., 2024 | 4 |
| [67] |  | Ferreira et al., 2018 | 4 |
| [68] |  | Ferreira et al., 2023 | 4 |
| [69] |  | Filgueiras et al., 2013 | 3 |
| [70] |  | Filipini, 2000 | 5 |
| [71] |  | Flôres et al., 2021 | 4 |
| [72] |  | Ford et al., 2025 | 6 |
| [73] |  | Fortunato et al., 2023 | 3 |
| [74] |  | Francisco et al., 2025 | 1 |
| [75] |  | Fuentefria et al., 2018 | 2 |
| [76] |  | Gastaud et al., 2023 | 1 |
| [77] |  | Gonçalves et al., 2019 | 4 |
| [78] |  | Gonçalves et al., 2025 | 1 |
| [79] |  | Goncharova et al., 2023 | 2 |
| [80] |  | Goulart Lemos et al., 2025 | 1 |
| [81] |  | Graminha et al., 1994 | 8 |
| [82] |  | Grantham-McGregor et al., 1998 | 1 |
| [83] |  | Guimarães et al., 2013 | 4 |
| [84] |  | Guimarães et al., 2022 | 3 |
| [85] |  | Halpern et al., 2008 | 6 |
| [86] |  | Hoffmann et al., 2022 | 5 |
| [87] |  | Isotani et al., 2009 | 4 |
| [88] |  | Israel and Yamaguchi, 2015 | 5 |
| [89] |  | Karam et al., 2016 | 2 |
| [90] |  | Kehdi et al., 2025 | 7 |
| [91] |  | Kofke et al., 2022 | 6 |
| [92] |  | Kwofie et al., 2025 | 1 |
| [93] |  | LaFreniere et al., 2002 | 3 |
| [94] |  | Leon et al., 2018 | 4 |
| [95] |  | Lima et al., 2004 | 1 |
| [96] |  | Lopes et al., 2009 | 1 |
| [97] |  | Lopez Boo et al., 2020 | 3 |
| [98] |  | Lordelo et al., 2006 | 1 |
| [99] |  | Machado et al., 2017 | 1 |
| [100] |  | Magalhães et al., 1989 | 9 |
| [101] |  | Maldonado-Martinez et al., 2025 | 1 |
| [102] |  | Maia et al., 2011 | 2 |
| [103] |  | Maia et al., 2012 | 5 |
| [104] |  | Mariano et al., 2021 | 5 |
| [105] |  | Marteleto et al., 2011 | 8 |
| [106] |  | Martineli et al., 2018 | 1 |
| [107] |  | Matijasevich et al., 2022 | 5 |
| [108] |  | McCormick et al., 2020 | 1 |
| [109] |  | McCoy et al., 2025 | 1 |
| [110] |  | Mélo et al., 2020 | 4 |
| [111] |  | Minami et al., 2001 | 4 |
| [112] |  | Montedori and Lima, 2024 | 1 |
| [113] |  | Morais et al., 2013 | 4 |
| [114] |  | Moreira et al., 2018 | 3 |
| [115] |  | Moreira et al., 2025 | 1 |
| [116] |  | Mota et al., 2020 | 1 |
| [117] |  | Moura et al., 2010 | 6 |
| [118] |  | Nobre et al., 2020 | 1 |
| [119] |  | Nogueira et al., 2015 | 1 |
| [120] |  | Novakoski et al., 2023 | 4 |
| [121] |  | Oliveira et al., 2012 | 4 |
| [122] |  | Oliveira, 2018 | 5 |
| [123] |  | Oliveira et al., 2022 | 2 |
| [124] |  | Oliveira et al., 2025 | 8 |
| [125] |  | Parada et al., 2024 | 8 |
| [126] |  | Paula et al., 2007 | 2 |
| [127] |  | Pedroso et al., 2021 | 1 |
| [128] |  | Pedrotti et al., 2024 | 1 |
| [129] |  | Pereira et al., 2016 | 1 |
| [130] |  | Pereira et al., 2021 | 6 |
| [131] |  | Petry et al., 2023 | 1 |
| [132] |  | Piccolo et al., 2016 | 2 |
| [133] |  | Procianoy et al., 2009 | 2 |
| [134] |  | Pureza et al., 2013 | 1 |
| [135] |  | Quevedo et al., 2012 | 1 |
| [136] |  | Ranquine et al., 2024 | 5 |
| [137] |  | Rebelo et al., 2024 | 5 |
| [138] |  | Renner et al., 2012 | 2 |
| [139] |  | Rezende et al., 2005 | 6 |
| [140] |  | Ribas and Kochen, 2016 | 7 |
| [141] |  | Ribeiro et al., 2014 | 6 |
| [142] |  | Ribeiro et al., 2025 | 1 |
| [143] |  | Ridgeway et al., 2024 | 1 |
| [144] |  | Righetto Greco et al., 2020 | 1 |
| [145] |  | Rocha et al. 2012 | 2 |
| [146] |  | Rocha et al., 2020 | 6 |
| [147] |  | Rocha et al., 2021 | 1 |
| [148] |  | Rodrigues et al., 2022 | 2 |
| [149] |  | Rubin et al., 2022 | 1 |
| [150] |  | Saad et al., 2017 | 2 |
| [151] |  | Saccani and Valentini, 2012 | 3 |
| [152] |  | Saccani and Valentini, 2013 | 4 |
| [153] |  | Saccani and Valentini, 2015 | 4 |
| [154] |  | Saccani et al., 2013 | 4 |
| [155] |  | Saccani et al., 2013 | 4 |
| [156] |  | Santos et al., 2000 | 1 |
| [157] |  | Santos et al., 2001 | 1 |
| [158] |  | Santos et al., 2013 | 1 |
| [159] |  | Santos et al., 2014 | 4 |
| [160] |  | Santos et al., 2015 | 9 |
| [161] |  | Santos et al., 2017 | 1 |
| [162] |  | Santos et al., 2024 | 1 |
| [163] |  | Schmidt et al., 2018 | 7 |
| [164] |  | Schoen-Ferreira et al., 2011 | 5 |
| [165] |  | Shavitt et al., 2022 | 3 |
| [166] |  | Silva et al., 2017 | 4 |
| [167] |  | Silva et al., 2018 | 1 |
| [168] |  | Silva et al., 2020 | 2 |
| [169] |  | Silva et al., 2024 | 6 |
| [170] |  | Silva et al., 2025 | 1 |
| [171] |  | Soares-Marangoni et al., 2025 | 5 |
| [172] |  | Solís-Cordero et al., 2020 | 4 |
| [173] |  | Sousa, 2020 | 5 |
| [174] |  | Souza et al., 2008 | 2 |
| [175] |  | Stasiak et al., 2014 | 1 |
| [176] |  | Tavares et al., 2021 | 1 |
| [177] |  | Teixeira et al., 2014 | 2 |
| [178] |  | Tovo-Rodrigues et al., 2020 | 6 |
| [179] |  | Valentini and Saccani, 2012 | 3 |
| [180] |  | Valentini et al., 2022 | 1 |
| [181] |  | Valentini, 2012 | 3 |
| [182] |  | Venancio et al., 2021 | 3 |
| [183] |  | Viegas da Silva et al., 2022 | 1 |
| [184] |  | Viegas da Silva et al., 2024 | 1 |
| [185] |  | Villar et al., 2018 | 3 |
| [186] |  | Villar et al., 2019 | 1 |
| [187] |  | Wehby and McCarthy, 2013 | 1 |
| [188] |  | Zago et al., 2023 | 1 |
| [189] |  | Zanella et al., 2021 | 3 |
| [190] |  | Zuanetti et al., 2021 | 2 |

*Legend: 1) Studies that did not present separate quantitative data regarding the prevalence of suspicion of developmental delay and/or behavior problem; 2) Studies with wrong population (like as adolescents, premature babies, children with high vulnerability, low birth weight babies and hospitalized children); 3) Instrument validation study; 4) Studies that did not use a screening test; 5) Publication type (conference abstracts, experimental studies, dissertations and theses); 6) Studies with duplicated data from another included study; 7) Another theme; 8) Full-text not available, even after trying to contact the corresponding authors (three attempts in a 3-week period); 9) Studies carried out in another country.

**References**

1. Almeida, K. M., Dutra, M. V. P., De Mello, R. R., Reis, A. B. R., & Martins, P. S. (2008). Concurrent validity and reliability of the Alberta Infant Motor Scale in premature infants. *Jornal de Pediatria*, *84*(5), 442–448. https://doi.org/10.2223/JPED.1836

2. Alvarenga, P. G., do Rosario, M. C., Cesar, R. C., Manfro, G. G., Moriyama, T. S., Bloch, M. H., Shavitt, R. G., Hoexter, M. Q., Coughlin, C. G., Leckman, J. F., & Miguel, E. C. (2016). Obsessive-compulsive symptoms are associated with psychiatric comorbidities, behavioral and clinical problems: a population-based study of Brazilian school children. *European Child & Adolescent Psychiatry*, *25*(2), 175–182. https://doi.org/10.1007/S00787-015-0723-3

3. Andrade, S. A., Santos, D. N., Bastos, A. C., Pedromônico, M. R. M., de Almeida-Filho, N., & Barreto, M. L. (2005). Family environment and child’s cognitive development: an epidemiological approach. *Revista de Saude Publica*, *39*(4), 606–611. https://doi.org/10.1590/S0034-89102005000400014

4. Anunciação, L., Squires, J., Clifford, J., & Landeira-Fernandez, J. (2019). Confirmatory analysis and normative tables for the Brazilian Ages and Stages Questionnaires: Social-Emotional. *Child: Care, Health and Development*, *45*(3), 387–393. https://doi.org/10.1111/CCH.12649

5. Ávila, C. N., Trettim, J. P., Rubin, B. B., Scholl, C. C., Coelho, F. T., de Matos, M. B., dos Santos Motta, J. V., Pinheiro, R. T., & de Avila Quevedo, L. (2025). Association between food insecurity and infant development at age 18 months in the urban area of Pelotas, Rio Grande do Sul State, Brazil. *Cadernos de Saude Publica*, *40*(12). https://doi.org/10.1590/0102-311XPT198023

6. Bandeira, M. de B., Rocha, S. S., de Souza, T. M. P., Del Prette, Z. A. P., & Del Prette, A. (2006). Behavior problems in elementary school children and their relation to social skills and academic performance. *Estudos de Psicologia (Natal)*, *11*(2), 199–208. https://doi.org/10.1590/S1413-294X2006000200009

7. Barros, A. J. D., Matijasevich, A., Santos, I. S., & Halpern, R. (2010). Child development in a birth cohort: effect of child stimulation is stronger in less educated mothers. *International Journal of Epidemiology*, *39*(1), 285. https://doi.org/10.1093/IJE/DYP272

8. Batista, C. L. C., & Brentani, A. V. M. (2023). Analysis of the influence of the timing of enrollment in daycares on child development. *Cadernos de Saude Publica*, *39*(4). https://doi.org/10.1590/0102-311XPT150622

9. Bertoldi, A. D., Rifas-Shiman, S. L., Boing, A. C., da Silva Dal Pizzol, T., Miranda, V. I. A., Silveira, M. P. T., Freitas Silveira, M., Domingues, M. R., Santos, I. S., Bassani, D. G., Tovo-Rodrigues, L., & Oken, E. (2020). Associations of acetaminophen use during pregnancy and the first year of life with neurodevelopment in early childhood. *Paediatric and Perinatal Epidemiology*, *34*(3), 267–277. https://doi.org/10.1111/PPE.12632

10. Beteli, V. C. (2006). *Acompanhamento do desenvolvimento infantil em creches* [Dissertação]. Universidade de São Paulo.

11. Bobbio, T. G., Morcillo, A. M., De Azevedo Barros Filho, A., & Gonçalves, V. M. G. (2007). Factors associated with inadequate fine motor skills in Brazilian students of different socioeconomic status. *Perceptual and Motor Skills*, *105*(3 Pt 2), 1187–1195. https://doi.org/10.2466/PMS.105.4.1187-1195

12. Bobbio, T. G., Gabbard, C., Gonçalves, V. G., Filho, A. A. B., & Morcillo, A. M. (2010). Interlimb coordination differentiates Brazilian children from two socioeconomic settings. *Pediatrics International : Official Journal of the Japan Pediatric Society*, *52*(3), 353–357. https://doi.org/10.1111/J.1442-200X.2009.02960.X

13. Bolsoni-Silva, A. T., & Loureiro, S. R. (2023). Associations between Sociodemographic and Behavioral Variables and Educational Practices on Mother-Child Dyads. *Acta Colombiana de Psicologia*, *26*(2), 11–22. https://doi.org/10.14718/ACP.2023.26.2.2

14. Bolsoni-Silva, A. T., Marturano, E. M., & Freiria, L. R. B. (2010). Indicativos de problemas de comportamento e de habilidades sociais em crianças: um estudo longitudinal. *Psicologia: Reflexão e Crítica*, *23*(3), 506–515. https://doi.org/10.1590/S0102-79722010000300011

15. Bolsoni-Silva, A. T., Loureiro, S. R., & Marturano, E. M. (2011). Problemas de comportamento e habilidades sociais infantis: modalidades de relato. *Psico*, *42*(3), 354–361. http://revistaseletronicas.pucrs.br/ojs/index.php/revistapsico/article/view/5813

16. Borsa, J. C., Souza, D. S. de, & Bandeira, D. R. (2011). Prevalência dos Problemas de Comportamento em uma Amostra de Crianças do Rio Grande do Sul. *Revista Psicologia: Teoria e Prática*, *13*(2), 15–29. https://editorarevistas.mackenzie.br/index.php/ptp/article/view/2821

17. Brentani, A., & Fink, G. (2016). Maternal depression and child development: Evidence from São Paulo’s Western Region Cohort Study. *Revista Da Associação Médica Brasileira*, *62*(6), 524–529. https://doi.org/10.1590/1806-9282.62.06.524

18. Brito, G. N. O., & De Onis, M. (2004). Growth status, behavior and neuropsychological performance: a study of Brazilian school age children. *Arquivos de Neuro-Psiquiatria*, *62*(4), 949–954. https://doi.org/10.1590/S0004-282X2004000600004

19. Brunstein, C., & Tiellet Nunes, M. L. (2003). *Visual attention and mother/child interaction: Early signs of the capacity to see the world*. https://www.researchgate.net/publication/286127666_Visual_attention_and_motherchild_interaction_Early_signs_of_the_capacity_to_see_the_world

20. Caçola, P., Gabbard, C., Santos, D. C. C., & Batistela, A. C. T. (2011). Development of the Affordances in the Home Environment for Motor Development-Infant Scale. *Pediatrics International : Official Journal of the Japan Pediatric Society*, *53*(6), 820–825. https://doi.org/10.1111/J.1442-200X.2011.03386.X

21. Caetano, S. C., Martins, S. S., Askari, M., Surkan, P., Sanchez, Z. M., Rosario, M. C., & Ribeiro, M. V. V. (2016). The Embu das Artes, Brazil, Preschool Mental Health Study : an epidemiological study of mental health and traumatic events: aims and methods. *Journal of the American Academy of Child & Adolescent Psychiatry*, *55*(10). https://doi.org/10.1016/J.JAAC.2016.09.053

22. Camargo-Figuera, F. A., Barros1, A. J. D., Santos, I. S., Matijasevich, A., & Barros, F. C. (2014). Early life determinants of low IQ at age 6 in children from the 2004 Pelotas Birth Cohort: a predictive approach. *BMC Pediatrics*, *14*(1). https://doi.org/10.1186/S12887-014-0308-1

23. Camargos, A. C. R., Mendonça, V. A., Andrade, C. A. de, Oliveira, K. S. C., & Lacerda, A. C. R. (2016). Overweight and obese infants present lower cognitive and motor development scores than normal-weight peers. *Research in Developmental Disabilities*, *59*, 410–416. https://doi.org/10.1016/j.ridd.2016.10.001

24. Campos, D., Gonçalves, V. M. G., Guerreiro, M. M., Santos, D. C., Goto, M. M. F., Arias, A. V., & Campos-Zanelli, T. M. (2012). Comparison of motor and cognitive performance in infants during the first year of life. *Pediatric Physical Therapy : The Official Publication of the Section on Pediatrics of the American Physical Therapy Association*, *24*(2), 193–197. https://doi.org/10.1097/PEP.0B013E31824D2DB7

25. Carvalho da Silva, R., Cursino, E. A., & Dias, L. B. da S. (1993). Acompanhamento do desenvolvimento psicomotor infantil em Unidades Básicas de Saúde. *Psicol. Teor. Pesqui*, 521–534.

26. Castelli, R. D., de Ávila Quevedo, L., da Cunha Coelho, F. M., Lopez, M. A., da Silva, R. A., Böhm, D. M., de Mattos Souza, L. D., de Matos, M. B., Pinheiro, K. A. T., & Pinheiro, R. T. (2015). Cognitive and language performance in children is associated with maternal social anxiety disorder: A study of young mothers in southern Brazil. *Early Human Development*, *91*(12), 707–711. https://doi.org/10.1016/J.EARLHUMDEV.2015.10.002

27. Castro, S. S., Machado, M. M. T., Correia, L. L., Farias-Antúnez, S., Lima, P. L. G. de S. B., Vasconcelos, S. C., Altafim, E. R. P., & Castro, M. C. (2025). Effect of sleep duration on child development in Fortaleza, Northeastern Brazil. *Jornal de Pediatria*, *101*(2), 238–246. https://doi.org/10.1016/j.jped.2024.09.002

28. Castro, S. S., Machado, M. M. T., Farias-Antúnez, S., Saldanha, A. B., Vasconcelos, S. C., Gomes, Y. V. C., Correia, L. L., & Castro, M. C. (2025). Association between early childhood development and shared play and reading. *Ciência & Saúde Coletiva*, *30*(5), e17272023. https://doi.org/10.1590/1413-81232025305.17272023

29. Cavaggioni, A. P. M., do Carmo Fernandes Martins, M., & Benincasa, M. B. (2020). Influence of type of birth on child development: a comparison by Bayley- III Scale. *Journal of Human Growth and Development*, *30*(2), 301–310. https://doi.org/10.7322/JHGD.V30.10382

30. Christovão, I. S., de Almeida Rodrigues, L., de Paula Machado, A. C. C., de Souza Pascoal, A. F., Fontes, D. E., Mendonça, K. T. de, de Castro Magalhães, L., & Camargos, A. C. R. (2024). Hybrid developmental follow-up for preterm infants in Brazil: A feasibility study. *Early Human Development*, *195*, 106069. https://doi.org/10.1016/J.EARLHUMDEV.2024.106069

31. Coelho, F. T., Trettim, J. P., Castelli, R. D., Soares, M. C., Dias, N. da C., Santo, G. C. D. E., Böhm, D. M., de Mello, D. B., Scholl, C. C., Rubin, B. B., de Matos, M. B., Pinheiro, R. T., & Quevedo, L. de A. (2021). Cognitive and Language Development in Preschoolers Is Related to Maternal Cognitive Performance: A Study of Young Mothers in an Urban Area of a City in Southern Brazil. *Interpersona: An International Journal on Personal Relationships*, *15*(2), 233–245. https://doi.org/10.5964/IJPR.4709

32. Costa, E. F., Cavalcante, L. I. C., Cruz, D. A., Frizzo, G. B., & Valente, M. D. R. (2021). Child naming skills and the quality of environments. *Psicologia: Teoria e Prática*, *23*(3), 1–25. https://doi.org/10.5935/1980-6906/EPTPHD13506

33. Cunha, H. L. da, & Melo, Á. N. de. (2008). *Avaliação do desenvolvimento neuropsicomotor em crianças na rede básica de saúde utilizando o teste de Denver II: identificação de fatores de risco materno* [Tese]. https://repositorio.ufrn.br/jspui/handle/123456789/13118

34. da Costa, A. C. R. V., Ferraz, N. N., & Berezovsky, A. (2021). Cognitive, motor, and visual development in healthy children in the first 42 months of life. *Arquivos Brasileiros de Oftalmologia*, *84*(5), 436–441. https://doi.org/10.5935/0004-2749.20210068

35. Da Fonseca Filho, G. G., Lopes, A. C., Bezerra, R. B., Candido, A. D. E. M., Arrais, N., Pereira, S. A., & Lindquist, A. R. (2021). Assessment of child development in premature babies based on the ICF biopsychosocial model. *European Journal of Physical and Rehabilitation Medicine*, *57*(4), 585–592. https://doi.org/10.23736/S1973-9087.20.06543-0

36. Da Silva, M. A., Filho, E. J. de M., & Bandeira, D. R. (2019). Development of the Dimensional Inventory of Child Development Assessment (IDADI). *Psico-USF*, *24*(1), 11–26. https://doi.org/10.1590/1413-82712019240102

37. Da Silva, M. A., Abrita, A. P. C. T., Filho, E. J. de M., & Bandeira, D. R. (2023). Associações do tipo de parto e amamentação com o desenvolvimento infantil. *Arquivos Brasileiros de Psicologia*, *75*(1), e002–e002. https://doi.org/10.36482/ARBP.V75I1.19343

38. da Silva, C. F. R., Greco, A. L. R., Castilho Cabrera Santos, D., Sgandurra, G., & Tudella, E. (2023). Association between Contextual Factors and Affordances in the Home Environment of Infants Exposed to Poverty. *Children (Basel, Switzerland)*, *10*(12). https://doi.org/10.3390/CHILDREN10121932

39. Almeida Filho, N. M. (1982). Estudo de prevalência de desordens mentais na infância e uma zona urbana de Salvador, Bahia. *Jornal Brasileiro de Psiquiatria*, *31*, 225–236.

40. De Andrade Leão, O. A., Bertoldi, A. D., Domingues, M. R., Murray, J., Santos, I. S., Barros, A. J. D., Matijasevich, A., & Mielke, G. I. (2024). Cross-sectional and prospective associations between screen time and childhood neurodevelopment in two Brazilian cohorts born 11 years apart. *Child: Care, Health and Development*, *50*(1). https://doi.org/10.1111/CCH.13165

41. De Borba, L. S., & Valentini, N. C. (2015). Motor and cognitive development of infants of adolescent and adult mothers: longitudinal study. *Revista Brasileira de Cineantropometria & Desempenho Humano*, *17*(4), 438–449. https://doi.org/10.5007/1980-0037.2015V17N4P438

42. De Mello, E. Q., Motta-Gallo, S., Goulart, F. C., Herrero, D., & Gallo, P. R. (2014). Motor development of Brazilian breastfeeding infants in socially unfavorable condition of life. *Journal of Human Growth and Development*, *24*(2), 163–167.

43. De Oliveira, S. R., Cabral, A. C., Machado, P., Jardim, J., Paulo, P., Paiva De Moraes, H., Juliana, M., Nahin, S., Novi, S. L., Mesquita, R. C., Marques, D. D. M., & Bouzada, M. C. F. (2017). Association between hemodynamic activity and motor performance in six-month-old full-term and preterm infants: a functional near-infrared spectroscopy study. *Neurophoton*, *5*(1), 11016. https://doi.org/10.1117/1.NPh.5.1.011016

44. De Oliveira, C. R., Moret-Tatay, C., & De Lima Argimon, I. I. (2021). Perfiles de Estrategias de Aprendizaje y Autoeficacia Académica en Estudiantes de Primaria. *Avaliação Psicológica*, *20*(4), 417–425. https://doi.org/10.15689/AP.2021.2004.22011.03

45. de Oliveira, I. P., Fernandéz, A. C., Salum, G. A., Gadelha, A., Pan, P. M., Miguel, E. C., Mograbi, D. C., & Bado, P. (2025). Longitudinal patterns of disordered eating behaviors in children and adolescents from the Brazilian High-Risk Cohort study for mental conditions. *Brazilian Journal of Psychiatry*, *47*, e20243867. https://doi.org/10.47626/1516-4446-2024-3867

46. Defilipo, É. C., Frônio, J. da S., Teixeira, M. T. B., Leite, I. C. G., Bastos, R. R., Vieira, M. de T., & Ribeiro, L. C. (2012). Oportunidades do ambiente domiciliar para o desenvolvimento motor. *Rev Saúde Pública*, *46*(4), 633–674. www.scielo.br/rsp

47. Defilipo, É. C., Magalhães, E. D. D., Máltar, C. M., de Oliveira, L. C., Neimerc, A. L. O., & Brugiolo, A. S. S. (2021). Home environment opportunities and motor development of infants in the first year. *Fisioterapia Em Movimento*, *34*, e34108. https://doi.org/10.1590/FM.2021.34108

48. Delgado, D. A., Michelon, R. C., Gerzson, L. R., Almeida, C. S. de, & Alexandre, M. da G. (2020). Avaliação do desenvolvimento motor infantil e sua associação com a vulnerabilidade social. *Fisioterapia e Pesquisa*, *27*(1), 48–56. https://doi.org/10.1590/1809-2950/18047027012020

49. Dias, J. A. B., De J. Manoel, E., Dias, R. B. de M., & Okazaki, V. H. A. (2013). Pilot study on infant swimming classes and early motor development. *Perceptual and Motor Skills*, *117*(3), 950–955. https://doi.org/10.2466/10.25.PMS.117X30Z2

50. Domingues, M. R., Matijasevich, A., Barros, A. J. D., Santos, I. S., Horta, B. L., & Hallal, P. C. (2014). Physical activity during pregnancy and offspring neurodevelopment and IQ in the first 4 years of life. *PloS One*, *9*(10). https://doi.org/10.1371/JOURNAL.PONE.0110050

51. Dornelas, L. F., Duarte, N. M. C., Morales, N. M. O., Pinto, R. M. C., Araújo, R. R. H., Pereira, S. A., & Magalhães, L. C. (2016). Functional Outcome of School Children With History of Global Developmental Delay. *Journal of Child Neurology*, *31*(8), 1041–1051. https://doi.org/10.1177/0883073816636224

52. Dos Santos Chiquetti, E. M., & Valentini, N. C. (2020). Test of Infant Motor Performance for Infants in Brazil: Unidimensional Model, Item Difficulty, and Motor Function. *Pediatric Physical Therapy : The Official Publication of the Section on Pediatrics of the American Physical Therapy Association*, *32*(4), 390–397. https://doi.org/10.1097/PEP.0000000000000745

53. dos Santos, V. A. P., Ferreira, L., Both, J., Caruzzo, N. M., & Vieira, J. L. L. (2020). Longitudinal study changes in coordination development disorder in preschool children. *Cadernos Brasileiros de Terapia Ocupacional*, *28*(4), 1180–1192. https://doi.org/10.4322/2526-8910.CTOAO2108

54. Dos Santos, R. S., de Lima Barreto, A. O. O., da Silva, M. C. C. P. B., Sá Nogueira, H. O. V., Peres, R. T., Ribeiro, M. G., & Ferreira, H. C. (2022). Agreement Between Alberta Infant Motor Scale Assessment and Maternal Perception of Motor Development in Full-Term Infants. *Developmental Neurorehabilitation*, *25*(6), 417–425. https://doi.org/10.1080/17518423.2022.2052373

55. Drachler, M. D. L., Marshall, T., & De Carvalho Leite, J. C. (2007). A continuous-scale measure of child development for population-based epidemiological surveys: a preliminary study using Item Response Theory for the Denver Test. *Paediatric and Perinatal Epidemiology*, *21*(2), 138–153. https://doi.org/10.1111/J.1365-3016.2007.00787.X

56. Duarte, M. G., Valentini, N. C., Nobre, G. C., & Benda, R. N. (2022). Contextual Factors and Motor Skills in Indigenous Amazon Forest and Urban Indigenous Children. *Frontiers in Public Health*, *10*, 858394. https://doi.org/10.3389/FPUBH.2022.858394/BIBTEX

57. Dumcke, T. S., Benedetti, A., Selistre, L. da S., Camardelo, A. M. P., & Silva, E. R. da. (2020). Associação entre exposição a resíduos urbanos e dificuldades emocionais e comportamentais de escolares. *Jornal de Pediatria*, *96*(3), 364–370. https://doi.org/10.1016/J.JPED.2018.11.014

58. Eickmann, S. H., Lima, A. C., Guerra, M. Q., Lima, M. C., Lira, P. I. C., Huttly, S. R. A., & Ashworth, A. (2003). Improved cognitive and motor development in a community-based intervention of psychosocial stimulation in northeast Brazil. *Developmental Medicine and Child Neurology*, *45*(8), 536–541. https://doi.org/10.1017/S0012162203000987

59. Eickmann, S. H., De Lira, P. I. C., Lima, M. D. C., Coutinho, S. B., Teixeira, M. D. L. P. D., & Ashworth, A. (2007). Breast feeding and mental and motor development at 12 months in a low-income population in northeast Brazil. *Paediatric and Perinatal Epidemiology*, *21*(2), 129–137. https://doi.org/10.1111/J.1365-3016.2007.00795.X

60. Emerich, D. R., da Rocha, M. M., Silvares, E. F. de M., & Gonçalves, J. de P. (2012). Diferenças Quanto ao Gênero entre Escolares Brasileiros Avaliados pelo Inventário de Comportamentos para Crianças e Adolescentes (CBCL/6-18). *PSICO*, *43*(3), 380–387.

61. Fattore, I. de M., Uhde, R. M., Oliveira, L. D., Roth, A. M., & Souza, A. P. R. de. (2017). Comparative analysis of initial vocalizations of preterm and full-term infants with and without risk for development. *CoDAS*, *29*(4), e20160075. https://doi.org/10.1590/2317-1782/20172016075

62. Fattore, I. de M., Uhde, R. M., Oliveira, L. D., Roth, A. M., & Souza, A. P. R. de. (2017). Análise comparativa das vocalizações iniciais de bebês prematuros e a termo, com e sem risco ao desenvolvimento. *CoDAS*, *29*(4), e20160075. https://doi.org/10.1590/2317-1782/20172016075

63. Fernandes, L. V., Goulart, A. L., Dos Santos, A. M. N., De Moraes Barros, M. C., Guerra, C. C., & Kopelman, B. I. (2012). Neurodevelopmental assessment of very low birth weight preterm infants at corrected age of 18-24 months by Bayley III scales. *Jornal de Pediatria*, *88*(6), 471–478. https://doi.org/10.2223/JPED.2230

64. Fernandes, M., Villar, J., Stein, A., Staines Urias, E., Garza, C., Victora, C. G., Barros, F. C., Bertino, E., Purwar, M., Carvalho, M., Giuliani, F., Wulff, K., Abubakar, A. A., Kihara, M., Cheikh Ismail, L., Aranzeta, L., Albernaz, E., Kunnawar, N., Di Nicola, P., … Kennedy, S. (2020). INTERGROWTH-21st Project international INTER-NDA standards for child development at 2 years of age: an international prospective population-based study. *BMJ Open*, *10*(6), e035258. https://doi.org/10.1136/BMJOPEN-2019-035258

65. Fernandes, M., Bassani, D., Albernaz, E., Bertoldi, A. D., Silveira, M. F., Matijsevich, A., Anselmi, L., Cruz, S., Halal, C. S., Tovo-Rodrigues, L., Cruz, G. I. N., Metgud, D., & Santos, I. S. (2022). Construction and validation of the Oxford Neurodevelopment Assessment (OX-NDA) in 1-year-old Brazilian children. *BMC Pediatrics*, *22*(1), 1–12. https://doi.org/10.1186/S12887-022-03794-1/TABLES/6

66. Fernandes, D. M. Z., & Lima, M. C. M. P. (2024). Child development: assessment of receptive and expressive language in preschoolers. *Estudos de Psicologia (Campinas)*, *41*, e210045. https://doi.org/10.1590/1982-0275202441E210045

67. Ferreira, L., Godinez, I., Gabbard, C., Vieira, J. L. L., & Caçola, P. (2018). Motor development in school-age children is associated with the home environment including socioeconomic status. *Child: Care, Health and Development*, *44*(6), 801–806. https://doi.org/10.1111/CCH.12606

68. Ferreira, L., de Fatima Matias de Souza, V., da Rocha, F. F., da Silva, P. N., Cheuczuk, F., & Lopes Vieira, J. L. (2023). Relationship between the sociodemographic profile and motor competence of children. *Cadernos Brasileiros de Terapia Ocupacional*, *31*, e3201. https://doi.org/10.1590/2526-8910.CTOAO244632011

69. Filgueiras, A., Pires, P., Maissonette, S., & Landeira-Fernandez, J. (2013). Psychometric properties of the Brazilian-adapted version of the Ages and Stages Questionnaire in public child daycare centers. *Early Human Development*, *89*(8), 561–576. https://doi.org/10.1016/j.earlhumdev.2013.02.005

70. Filipini, R., & Siqueira, A. A. F. de. (2000). *Crescimento e Desenvolvimento de Crianças Residentes em uma favela do Município de São Paulo* [Tese]. Universidade de São Paulo.

71. Flôres, F. S., Rodrigues, L. P., & Cordovil, R. (2021). Relationship between the Affordances for Motor Behavior of Schoolchildren (AMBS) and Motor Competence Assessment (MCA) in Brazilian Children. *Children 2021, Vol. 8, Page 705*, *8*(8), 705. https://doi.org/10.3390/CHILDREN8080705

72. Ford, L. A., Buccini, G., Saragosa, A. C., Martins, I. de A., Moreira, J. M., Lemos, S. M. A., Alves, C. R. L., & Mara Gonçalves de Oliveira Azevedo, V. (2025). Exclusive breastfeeding modifies the association between maternal education and child development: a cross-sectional study nested in a cohort. *Jornal de Pediatria*, *101*(4), 511–519. https://doi.org/10.1016/J.JPED.2025.02.004

73. Fortunato, C. A. Q., de Lima Isaac, M., Angelo Hyppolito, M., Fortunato Queiroz, C., de Lima Isaac, M., & Angelo Hyppolito, M. (2023). Cross-cultural adaptation of the new Reynell developmental language scales to Brazilian Portuguese. *Brazilian Journal of Otorhinolaryngology*, *89*(6), 101332. https://doi.org/10.1016/J.BJORL.2023.101332

74. Francisco, A. S. P. G., Graciosa, M. D., Pacheco, S. C. da S., & Sanada, L. S. (2025). Gross motor trajectories of pre-term and full-term infants under different parental educational approaches. *Journal of Child Health Care : For Professionals Working with Children in the Hospital and Community*, *29*(2), 408–423. https://doi.org/10.1177/13674935231211954

75. Fuentefria, R. N., Silveira, R. C., & Procianoy, R. S. (2018). Neurodevelopment and Growth of a Cohort of Very Low Birth Weight Preterm Infants Compared to Full-Term Infants in Brazil. *American Journal of Perinatology*, *35*(2), 152–162. https://doi.org/10.1055/S-0037-1606351

76. Gastaud, L. M., Trettim, J. P., Scholl, C. C., Rubin, B. B., Coelho, F. T., Krause, G. B., Ferreira, N. M., de Matos, M. B., Pinheiro, R. T., & de Avila Quevedo, L. (2023). Screen time: Implications for early childhood cognitive development. *Early Human Development*, *183*. https://doi.org/10.1016/j.earlhumdev.2023.105792

77. Gonçalves, T. R., Duku, E., & Janus, M. (2019). Developmental health in the context of an early childhood program in Brazil: the “Primeira Infância Melhor” experience. *Cadernos de Saúde Pública*, *35*(3), e00224317. https://doi.org/10.1590/0102-311X00224317

78. Gonçalves, H., Soares, P. S. M., da Silva Dias, M., Alves, E. D., Lessa, I. M., Danigno, J. F., Brina, K. R., Goularte, L. M., da Silva, L. S., Cleff, M. M., Menezes, A. M. B., & Wehrmeister, F. C. (2025). Does maternal anxiety predict child behavioral problems? An examination of internalizing and externalizing behaviors in children of participants in a Brazilian cohort study. *Journal of Affective Disorders*, *376*, 366–375. https://doi.org/10.1016/J.JAD.2025.02.037

79. Goncharova, Y., & Ross, J. (2023). The self-concept questionnaire offers a multidimensional, developmentally sensitive measure of the capacity for self-reflection in young children. *Infant and Child Development*, *32*(6), e2459. https://doi.org/10.1002/ICD.2459

80. Goulart Lemos, N. B. A., Carson, V., da Silva Santos, P. G., de Aguiar Lemos, F., Duncan, M., & de Lucena Martins, C. M. (2025). Adherence to the 24-h Movement Behaviors Guidelines and Associations With Cognitive and Behavioral Self-Regulation Among Brazilian Preschoolers. *American Journal of Human Biology : The Official Journal of the Human Biology Council*, *37*(1). https://doi.org/10.1002/AJHB.24206

81. Graminha, S. S. V. (1994). Problemas emocionais comportamentais em uma amostra de escolares: incidência em função do sexo e idade. *Psico*, *25*, 44–74.

82. Grantham-McGregor, S. M., Lira, P. I. C., Ashworth, A., Morris, S. S., & Assuncao, A. M. S. (1998). The development of low birth weight term infants and the effects of the environment in Northeast Brazil. *Journal of Pediatrics*, *132*(4), 661–666. https://doi.org/10.1016/S0022-3476(98)70357-9

83. Guimarães, A. F., de Carvalho, D. V., Machado, N. Á. A., Baptista, R. A. N., & Lemos, S. M. A. (2013). Risk of developmental delay of children aged between two                and 24 months and its association with the quality of family stimulus. *Revista Paulista de Pediatria*, *31*(4), 452–458. https://doi.org/10.1590/S0103-05822013000400006

84. Guimarães, M. A. P., Magalhães, L. D. C., Moreira, R. S., Bessa, F. R., & Alves, C. R. L. (2022). Survey of Well-Being of Young Children (SWYC): Preliminary Norms for Screening for Developmental Delay in Brazilian Children Younger than 65 Months. *Journal of Developmental and Behavioral Pediatrics : JDBP*, *43*(9), E614–E622. https://doi.org/10.1097/DBP.0000000000001133

85. Halpern, R., Barros, A. J. D., Matijasevich, A., Santos, I. S., Victora, C. G., & Barros, F. C. (2008). Developmental status at age 12 months according to birth weight and family income: a comparison of two Brazilian birth cohorts. *Cadernos de Saúde Pública*, *24*(SUPPL.3), s444–s450. https://doi.org/10.1590/S0102-311X2008001500010

86. Hoffmann, E. V., Duarte, C. S., Matsuzaka, C. T., Milani, A. C. C., Fossaluza, V., Mello, A. F., & Mello, M. F. (2022). The positive impact of an intervention for maternal depression on child emotional and behavioral symptoms in a low-resource setting. *Brazilian Journal of Psychiatry*, *44*(6), 590–601. https://doi.org/10.47626/1516-4446-2022-2498

87. Isotani, S. M., de Azevedo, M. F., Chiari, B. M., & Perissinoto, J. (2009). Expressive language of two year-old pre-term and full-term children. *Pro-Fono : Revista de Atualizacao Cientifica*, *21*(2), 155–160. https://doi.org/10.1590/S0104-56872009000200012

88. Israel, V., & Yamaguchi, B. (2015). Physical therapy assessment of psychomotor and motor skills of children using Denver II. *Physiotherapy*, *101*, e655–e656. https://doi.org/10.1016/j.physio.2015.03.3491

89. Karam, S. M., Barros, A. J. D., Matijasevich, A., Dos Santos, I. S., Anselmi, L., Barros, F., Leistner-Segal, S., Félix, T. M., Riegel, M., Maluf, S. W., Giugliani, R., & Black, M. M. (2016). Intellectual Disability in a Birth Cohort: Prevalence, Etiology, and Determinants at the Age of 4 Years. *Public Health Genomics*, *19*(5), 290–297. https://doi.org/10.1159/000448912

90. Kehdi, R. C., Silva, M. F. S., Cavalcante, L. R. L., Fiorenza, N. G., Viana, M., Leite, I. B., dos Santos Silva, B. R., de Assis, D. F., Cortez, P. C., Bezerra, D. L., Motta, P. C., Marques, J. A. L., Chaves Filho, A. J. M., Correia, É., Farias, A. A., Miyajima, F., Teixeira, A. L., Thomason, M. E., Carvalho, F. H. C., & Macedo, D. S. (2025). Cord blood cytokines/chemokines linked to delays in toddlers exposed to SARS-CoV-2 prenatally. *Pediatric Research*, 1–10. https://doi.org/10.1038/S41390-025-04192-W;KWRD

91. Kofke, L., Pérez-Escamilla, R., Gubert, M. B., & Buccini, G. (2022). Socio-demographic, maternal, and infant characteristics associated with early childhood development delays among children of young mothers in Brasília, Brazil. *PloS One*, *17*(3). https://doi.org/10.1371/JOURNAL.PONE.0266018

92. Kwofie, N. A., Suherman, A., Florindo, A. A., Staiano, A., Amy S., H. A., Okely, A. D., Hamdouchi, A. E., Pham, B. N., Draper, C. E., Tanaka, C., Koh, D., Kim, D. H., Webster, E. K., Guan, H., Tang, H. K., Reilly, J. J., Chong, K. H., Löf, M., Tremblay, M. S., … Janssen, X. (2025). Associations Between Sedentary Behaviour and Fine and Gross Motor Skills in 3‐ to 4‐Year‐Olds: A Secondary Data Analysis From Sunrise International Study Pilot Studies. *Child*, *51*(3), e70092. https://doi.org/10.1111/CCH.70092

93. Lafreniere, P., Masataka, N., Butovskaya, M., Dessen, M. A., Chen, Q., Atwanger, K., Schreiner, S., Montirosso, R., & Frigerio, A. (2002). Cross-cultural analysis of social competence and behavior problems in preschoolers. *Early Education and Development*, *13*(2), 187–199. https://doi.org/10.1207/S15566935EED1302_6

94. Leon, C. B. R., Dias, N. M., Martins, G. L. L., & Seabra, A. G. (2018). Executive functions in preschool children: development and relationships with language and behavior. *Psicologia: Teoria e Prática*, *20*(3), 121-137. https://doi.org/10.5935/1980-6906/PSICOLOGIA.V20N3P121-137

95. Lima, M. C., Eickmann, S. H., Lima, A. C. V., Guerra, M. Q., Lira, P. I. C., Huttly, S. R. A., & Ashworth, A. (2004). Determinants of mental and motor development at 12 months in a low income population: a cohort study in northeast Brazil. *Acta Paediatrica (Oslo, Norway : 1992)*, *93*(7), 969–975. https://doi.org/10.1111/J.1651-2227.2004.TB18257.X

96. Lopes, V. B., De Lima, C. D., & Tudella, E. (2009). Motor acquisition rate in Brazilian infants. *Infant and Child Development*, *18*(2), 122–132. https://doi.org/10.1002/ICD.595

97. Lopez Boo, F., Cubides Mateus, M., & Llonch Sabatés, A. (2020). Initial psychometric properties of the Denver II in a sample from Northeast Brazil. *Infant Behavior and Development*, *58*, 101391. https://doi.org/10.1016/J.INFBEH.2019.101391

98. Lordelo, E. da R., da França, C. B., Lopes, L. M. dos S., Dacal, M. del P. O., Carvalho, C. S., Guirra, R. C., & Chalub, A. A. (2006). Investimento parental e desenvolvimento da criança. *Estudos de Psicologia (Natal)*, *11*(3), 257–264. https://doi.org/10.1590/S1413-294X2006000300002

99. Machado, D., Guidony Pereira, K. R., Müller, A. B., & Valentini, N. C. (2017). Desenvolvimento motor, cognição e linguagem em lactentes que frequentam creches. *Scientia Medica*, *27*(4), ID27993–ID27993. https://doi.org/10.15448/1980-6108.2017.4.27993

100. Magalhaes, L. C., Koomar, J. A., & Cermak, S. A. (1989). Bilateral motor coordination in 5- to 9-year-old children: a pilot study. *The American Journal of Occupational Therapy : Official Publication of the American Occupational Therapy Association*, *43*(7), 437–443. https://doi.org/10.5014/AJOT.43.7.437

101. Maldonado-Martinez, A., Caetano, S. C., Ribeiro, M. V., Restrepo-Henao, A., Okuda, P. M., Fidalgo, T. M., Surkan, P. J., Silva, L. M., & Martins, S. S. (2025). Internalizing problems can differ in boys and girls since early childhood: findings from the Child Behavioral Checklist 1.5-5. *Brazilian Journal of Psychiatry*, *47*, e20243616. https://doi.org/10.47626/1516-4446-2024-3616

102. Maia, P. C., Silva, L. P., Oliveira, M. M. C., & Cardoso, M. V. L. M. L. (2011). Motor development of preterm and term infants-using the Alberta Infant Motor Scale. *Acta Paul Enferm*, *24*(5), 670–675.

103. Maia, M., Figueiredo, F. P., Barbieri, M. A., Silva, A. A. M., Loureiro, S. R., & Bettiol, H. (2012). Prolonged non-nutritive suckling habits are associated with emotional and behavioral problems in school-age children. *European Psychiatry*, *27*, 1. https://doi.org/10.1016/S0924-9338(12)74448-6

104. Mariano, M., da Silva, A. R., Lima, J. L. S., de Pinho, N. T., Cogo-Moreira, H., Melo, M. H. S., Mari, J. J., Sanchez, Z. M., & Caetano, S. C. (2021). Effectiveness of the Elos 2.0 prevention programme for the reduction of problem behaviours and promotion of social skills in schoolchildren: study protocol for a cluster-randomized controlled trial. *Trials*, *22*(1), 1–11. https://doi.org/10.1186/S13063-021-05408-0/PEER-REVIEW

105. Marteleto, M., Silva, D., Cabral, M., Leite, N., Machado, R., & Luna, R. M. (2011). Behavioral problems and atypical behavior in children and adolescents. *Pediatr. Res.*, *69*(4), 367.

106. Martineli, A. K. B., Pizeta, F. A., & Loureiro, S. R. (2018). Behavioral problems of school children: impact of social vulnerability, chronic adversity, and maternal depression. *Psicologia: Reflexao e Critica*, *31*(1), 1–11. https://doi.org/10.1186/S41155-018-0089-9/TABLES/3

107. Matijasevich, A., Cardoso, M., Santos, I., Barros, A., & Tovo-Rodrigues, L. (2022). Early child development: findings and perspectives from birth cohorts in Brazil. *Archives of Disease in Childhood*, *107*(Suppl 1), A3–A3. https://doi.org/10.1136/ARCHDISCHILD-2022-ICHGC.4

108. McCormick, B. J. J., Caulfield, L. E., Richard, S. A., Pendergast, L., Seidman, J. C., Maphula, A., Koshy, B., Blacy, L., Roshan, R., Nahar, B., Shrestha, R., Rasheed, M., Svensen, E., Rasmussen, Z., Scharf, R. J., Haque, S., Oria, R., Murray-Kolb, L. E., Acosta, A. M., … Strand, T. (2020). Early Life Experiences and Trajectories of Cognitive Development. *Pediatrics*, *146*(3). https://doi.org/10.1542/PEDS.2019-3660

109. McCoy, D. C., Hanno, E. C., Tan, F., Fonseca, G., Ponczek, V. P., & Pinto, C. C. de X. (2025). Exploring the Effects of a Social-Emotional Learning Intervention in Brazilian Primary Schools: Findings from Year Two of Implementation. *Journal of Research on Educational Effectiveness*, *18*(3), 588–614. https://doi.org/10.1080/19345747.2024.2322609

110. Mélo, T. R., de Araujo, L. B., Yamaguchi, B., Ferreira, M. de P., & Israel, V. L. (2020). Quality of life and neuropsychomotor development of infants between 4-18 months in daycare center. *Ciencia & Saude Coletiva*, *25*(8), 3175–3184. https://doi.org/10.1590/1413-81232020258.21002018

111. Minami, M. A. Y., Funayama, C. A. R., & Daneluzzi, J. C. (2001). Focusing on dissociated motor development in Brazilian children. *Arquivos de Neuro-Psiquiatria*, *59*(3-B), 686–690. https://doi.org/10.1590/S0004-282X2001000500006

112. Montedori, K. T., & Lima, M. C. M. P. (2024). Early childhood development monitoring during the first thousand days: Investigating the relationship between the developmental surveillance instrument and standardized scales. *Early Human Development*, *190*. https://doi.org/10.1016/j.earlhumdev.2024.105965

113. Morais, M. de L. S. e, Lucci, T. K., & Otta, E. (2013). Postpartum depression and child development in first year of life. *Estudos de Psicologia (Campinas)*, *30*(1), 7–17. https://doi.org/10.1590/S0103-166X2013000100002

114. Moreira, R. S., Magalhães, L. C., Siqueira, C. M., & Alves, C. R. L. (2018). “Survey of Wellbeing of Young Children (SWYC)”: how does it fit for screening developmental delay in Brazilian children aged 4 to 58 months? *Research in Developmental Disabilities*, *78*, 78–88. https://doi.org/10.1016/j.ridd.2018.05.003

115. Moreira, R. S., Guimarães, M. A. P., Magalhães, L. de C., Moreira, J. M., & Alves, C. R. L. (2025). Early symptoms of autism spectrum disorders and association with Brazilian children’s development and behavior. *CoDAS*, *37*(4), e20240306. https://doi.org/10.1590/2317-1782/E20240306EN

116. Mota, J. G., Clark, C. C. T., Bezerra, T. A., Lemos, L., Reuter, C. P., Mota, J. A. P. S., Duncan, M. J., & Martins, C. M. D. L. (2020). Twenty-four-hour movement behaviours and fundamental movement skills in preschool children: A compositional and isotemporal substitution analysis. *Journal of Sports Sciences*, *38*(18), 2071–2079. https://doi.org/10.1080/02640414.2020.1770415

117. De Moura, D. R., Costa, J. C., Santos, I. S., Barros, A. J. D., Matijasevich, A., Halpern, R., Dumith, S., Karam, S., & Barros, F. C. (2010). Risk factors for suspected developmental delay at age 2 years in a Brazilian birth cohort. *Paediatric and Perinatal Epidemiology*, *24*(3), 211–221. https://doi.org/10.1111/J.1365-3016.2010.01115.X

118. Nobre, J. N. P., Vinolas Prat, B., Santos, J. N., Santos, L. R., Pereira, L., Guedes, S. da C., Ribeiro, R. F., & Morais, R. L. de S. (2020). Quality of interactive media use in early childhood and child development: a multicriteria analysis^,^. *Jornal de Pediatria*, *96*(3), 310–317. https://doi.org/10.1016/J.JPED.2018.11.015

119. Nogueira, S. F., Figueiredo, E. M., Gonçalves, R. V., & Mancini, M. C. (2015). Relation between hand function and gross motor function in full term                infants aged 4 to 8 months. *Brazilian Journal of Physical Therapy*, *19*(1), 52–60. https://doi.org/10.1590/BJPT-RBF.2014.0070

120. Novakoski, K. R. M., Araujo, L. B. de, Mélo, T. R., & Israel, V. L. (2023). Mom Didn’t Go to School, Dad Is Out of Work: Associations between Maternal Educational Attainment, Family Socioeconomic Status, and Infant Development. *Health Services Insights*, *16*. https://doi.org/10.1177/11786329231163009

121. Oliveira, L. L., Costa, V. M. R., Requeijo, M. R., Rebolledo, R. S., de Fátima Pimenta, A., & Lemos, S. M. A. (2012). Desenvolvimento infantil: concordância entre a caderneta de saúde da criança e o manual para vigilância do desenvolvimento infantil [Tese]. In *Revista Paulista de Pediatria* (Vol. 30, Issue 4). https://doi.org/10.1590/S0103-05822012000400004

122. Oliveira, C. V. R. de. (2018). *Avaliação do desenvolvimento infantil com foco nos determinantes sociais* [Universidade de São Paulo]. https://doi.org/10.11606/T.7.2018.TDE-05112018-132533

123. Oliveira, T. D. O., Costa, D. S., Alvim-Soares, A., de Paula, J. J., Kestelman, I., Silva, A. G., Malloy-Diniz, L. F., & Miranda, D. M. (2022). Children’s behavioral problems, screen time, and sleep problems’ association with negative and positive parenting strategies during the COVID-19 outbreak in Brazil. *Child Abuse & Neglect*, *130*(Pt 1). https://doi.org/10.1016/J.CHIABU.2021.105345

124. de Oliveira, C. V. R., Bordignon, S. S., Martins, R. C., Blumenberg, C., Martins-Silva, T., Costa, F., Cesar, J., Springer, P., & de Mola, C. L. (2025). Trajectories of maternal depression, anxiety, stress, and child developmental milestones at 24 months. *Journal of Child and Adolescent Mental Health*. https://doi.org/10.2989/17280583.2025.2452533

125. Parada, A. P., de Rezende, M. G., Figueiredo de Paula Eduardo, J. A., de Figueiredo, F. P., Bettiol, H., Carvalho-Cavalli, R., Cardoso, V. C., & Del Ben, C. M. (2024). Associações entre depressão materna e o desenvolvimento neurocomportamental das crianças: Um estudo longitudinal e descritivo. *Prometeica*, *30*, 236–251. https://doi.org/10.34024/prometeica.2024.30.18857

126. Paula, C. S., Duarte, C. S., & Bordin, I. A. S. (2007). Prevalence of mental health problems in children and adolescents from the outskirts of Sao Paulo City: treatment needs and service capacity evaluation. *Brazilian Journal of Psychiatry*, *29*(1), 11–17. https://doi.org/10.1590/S1516-44462006005000012

127. Pedroso, J., Herkrath, F. J., Buccini, G., Venancio, S. I., Pérez-Escamilla, R., & Gubert, M. B. (2021). Maternal Mental Health Mediates the Relationship Between Household Food Insecurity and Early Child Development in Brazil. *Food and Nutrition Bulletin*, *42*(1), 77–90. https://doi.org/10.1177/0379572120987815

128. Pedrotti, B. G., Bandeira, D. R., & Frizzo, G. B. (2024). Context of digital media use in early childhood: Factors associated with cognitive development up to 36 months of age. *Infant Behavior and Development*, *76*, 101963. https://doi.org/10.1016/J.INFBEH.2024.101963

129. Pereira, K. R. G., Valentini, N. C., & Saccani, R. (2016). Brazilian infant motor and cognitive development: Longitudinal influence of risk factors. *Pediatrics International : Official Journal of the Japan Pediatric Society*, *58*(12), 1297–1306. https://doi.org/10.1111/PED.13021

130. Pereira, L., da Conceição Guedes, S., de Souza Morais, R. L., Nobre, J. N. P., & Santos, J. N. (2021). Environmental resources, types of toys, and family practices that enhance child cognitive development. *CoDAS*, *33*(2), 1–8. https://doi.org/10.1590/2317-1782/20202019128

131. Petry, I., Trettim, J. P., de Matos, M. B., Rubin, B. B., Scholl, C. C., Firpo, P. de O., Pinheiro, K. A. T., Quevedo, L. de A., Ghisleni, G. C., Tavares Pinheiro, R., & Motta, J. V. dos S. (2023). Breastfeeding and its relationship with motor development of infants at 3–4 months of age. *Early Child Development and Care*, *193*(2), 211–222. https://doi.org/10.1080/03004430.2022.2075355

132. Piccolo, L. da R., Segabinazi, J. D., Falceto, O. G., Fernandes, C. L. C., Bandeira, D. R., Trentini, C. M., Hutz, C. S., & Salles, J. F. (2016). Developmental delay in early childhood is associated with visual-constructive skills at school age in a Brazilian cohort. *Psicologia: Reflexão e Crítica*, *29*(1), 41. https://doi.org/10.1186/S41155-016-0048-2

133. Procianoy, R. S., Koch, M. S., & Silveira, R. C. (2009). Neurodevelopmental outcome of appropriate and small for gestational age very low birth weight infants. *Journal of Child Neurology*, *24*(7), 788–794. https://doi.org/10.1177/0883073808331087

134. Pureza, J. R., Gonçalves, H. A., Branco, L., Grassi-Oliveira, R., & Paz Fonseca, R. (2013). Executive functions in late childhood: age differences among groups. *Psychology & Neuroscience*, *6*(1), 79–88. https://doi.org/10.3922/J.PSNS.2013.1.12

135. Quevedo, L. A., Silva, R. A., Godoy, R., Jansen, K., Matos, M. B., Tavares Pinheiro, K. A., & Pinheiro, R. T. (2012). The impact of maternal post-partum depression on the language development of children at 12 months. *Child: Care, Health and Development*, *38*(3), 420–424. https://doi.org/10.1111/J.1365-2214.2011.01251.X

136. Ranquine, L. G., Barbalho, B. O., Ferreira, C. S., Ribas, M. T. N., Moura, C. O., Bissoli, M. T. M., Torres, A. G., F, R., & El-Bacha, T. (2024). Vitamin D metabolites and oxidized polyunsaturated fatty acids in plasma and human milk: Implications for the metabolic and mental health of the mother and infant development in the first six months postpartum. *Placenta*, *153*, e38–e39. https://doi.org/10.1016/J.PLACENTA.2024.05.121

137. Rebelo, F., de Moura, C. O., Ranquine, L. G., de Mattos Teixeira, T., Ribas, M. T. N., Vitorino, R. S., de Abranches, A. D., Costa, R. P., de Britto, J. A. A., Marano, D., Soares, F. V. M., de Fátima Junqueira- Marinho, M., de Sousa, C. A. M., Franco-Sena, A. B., Nardi, A. E., El-Bacha, T., & Moreira, M. E. L. (2024). Plasma and breast milk adipokines in women across the first year postpartum and their association with maternal depressive symptoms and infant neurodevelopment: Protocol for the APPLE prospective cohort study. *PloS One*, *19*(10). https://doi.org/10.1371/JOURNAL.PONE.0310847

138. Renner, A. C., Da Silva, A. A. M., Rodriguez, J. D. M., Simões, V. M. F., Barbieri, M. A., Bettiol, H., Thomaz, E. B. A. F., & Da Conceiçaão Saraiva, M. (2012). Are mental health problems and depression associated with bruxism in children? *Community Dentistry and Oral Epidemiology*, *40*(3), 277–287. https://doi.org/10.1111/J.1600-0528.2011.00644.X

139. Rezende, M. A., Beteli, V. C., & dos Santos, J. L. F. (2005). Follow-up of the child’s motor abilities in day-care centers and pre-schools. *Revista Latino-Americana de Enfermagem*, *13*(5), 619–625. https://doi.org/10.1590/S0104-11692005000500003

140. Ribas, A., & Kochen, A. P. (2016). Brazilian scale of hearing and language development in children (EDAL-1) with cochlear implant and less than two years of hearing AGE Abstract. *International Tinnitus Journal*, *20*(1), 7–10. https://doi.org/10.5935/0946-5448.20160002

141. Ribeiro, D. G., Perosa, G. B., & Padovani, F. H. P. (2014). Mental Health, Mother-Child Interaction and Development at the End of the First Year of Life^1^. *Paidéia (Ribeirão Preto)*, *24*(59), 331–339. https://doi.org/10.1590/1982-43272459201407

142. Ribeiro, M. V. V., Muszkat, D., Fidalgo, T. M., Mariano, M., Rosário, M. C. do, Rocca, C. C. A., Surkan, P. J., Martins, S. S., Okuda, P. M. M., & Caetano, S. C. (2025). Is school readiness associated with externalizing symptoms? *Early Child Development and Care*. https://doi.org/10.1080/03004430.2025.2546146

143. Ridgeway, K., Park, S., Okuda, P. M. M., Félix, E., Ribeiro, M., Martins, S. S., Caetano, S. C., & Surkan, P. J. (2024). Caregiver Parenting Stress Associated with Delays in Child Social-Emotional and Motor Development. *Journal of Child and Family Studies*, *33*(6), 1921–1933. https://doi.org/10.1007/S10826-024-02787-1/METRICS

144. Righetto Greco, A. L., Sato, N. T. da S., Cazotti, A. M., & Tudella, E. (2020). Is Segmental Trunk Control Related to Gross Motor Performance in Healthy Preterm and Full-Term Infants? *Journal of Motor Behavior*, *52*(6), 666–675. https://doi.org/10.1080/00222895.2019.1673694

145. Rocha, M. M., Rescorla, L. A., Emerich, D. R., Silvares, E. F. M., Borsa, J. C., Araújo, L. G. S., Bertolla, M. H. S. M., Oliveira, M. S., Perez, N. C. S., Freitas, P. M., & Assis, S. G. (2012). Behavioural/emotional problems in Brazilian children: findings from parents’ reports on the Child Behavior Checklist. *Epidemiology and Psychiatric Sciences*, *22*(4), 329. https://doi.org/10.1017/S2045796012000637

146. Rocha, P. R. H., Saraiva, M. da C. P., Barbieri, M. A., Ferraro, A. A., & Bettiol, H. (2020). Association of preterm birth and intrauterine growth restriction with childhood motor development: Brisa cohort, Brazil. *Infant Behavior & Development*, *58*. https://doi.org/10.1016/J.INFBEH.2020.101429

147. Rocha, H. A. L., Correia, L. L., Leite, Á. J. M., Machado, M. M. T., Lindsay, A. C., Rocha, S. G. M. O., Campos, J. S., Cavalcante e Silva, A., & Sudfeld, C. R. (2021). Screen time and early childhood development in Ceará, Brazil: a population-based study. *BMC Public Health*, *21*(1), 1–8. https://doi.org/10.1186/S12889-021-12136-2/FIGURES/1

148. Rodrigues, J. C. de L., Gaspardo, C. M., & Linhares, M. B. M. (2022). Development of neonatal high-risk preterm infants in comparison to full-term counterparts. *Applied Neuropsychology. Child*, *11*(4), 840–849. https://doi.org/10.1080/21622965.2021.1988601

149. Rubin, B. B., Trettim, J. P., Scholl, C. C., Coelho, F. T., Puccinelli, E. F., de Matos, M. B., Ghisleni, G., Pinheiro, R. T., & Quevedo, L. de A. (2022). Maternal-Fetal Attachment and Social-Emotional Development in Infants at 3 Months of Age: A Population-Based Study in Southern Brazil. *Interpersona: An International Journal on Personal Relationships*, *16*(2), 260–276. https://doi.org/10.5964/IJPR.6693

150. Saad, L. O., Do Rosario, M. C., Cesar, R. C., Batistuzzo, M. C., Hoexter, M. Q., Manfro, G. G., Shavitt, R. G., Leckman, J. F., Miguel, E. C., & Alvarenga, P. G. (2017). The Child Behavior Checklist-Obsessive-Compulsive Subscale Detects Severe Psychopathology and Behavioral Problems Among School-Aged Children. *Journal of Child and Adolescent Psychopharmacology*, *27*(4), 342–348. https://doi.org/10.1089/CAP.2016.0125

151. Saccani, R., & Valentini, N. C. (2012). Reference curves for the Brazilian Alberta Infant Motor Scale: percentiles for clinical description and follow-up over time. *Jornal de Pediatria*, *88*(1), 40–47. https://doi.org/10.2223/JPED.2142

152. Saccani, R., & Valentini, N. C. (2013). Cross-cultural analysis of the motor development of      Brazilian, Greek and Canadian infants assessed with the Alberta Infant Motor      Scale. *Revista Paulista de Pediatria*, *31*(3), 350–358. https://doi.org/10.1590/S0103-05822013000300012

153. Saccani, R., & Valentini, N. C. (2015). Postural control in children born at term according to the alberta infant motor scale: comparison between sexes. *Journal of Human Growth and Development*, *25*(3), 364–370. https://doi.org/10.7322/JHGD.106014

154. Saccani, R., Valentini, N. C., Venturella, C. B., & Zanandrea, G. (2013). Motor development of children between 0 and 18 months of age:  Differences between sexes. *Motricidade*, *9*(2), 3–12. https://doi.org/10.6063/MOTRICIDADE.617

155. Saccani, R., Valentini, N. C., Pereira, K. R., Müller, A. B., & Gabbard, C. (2013). Associations of biological factors and affordances in the home with infant motor development. *Pediatrics International : Official Journal of the Japan Pediatric Society*, *55*(2), 197–203. https://doi.org/10.1111/PED.12042

156. Santos, D. C. C., Gabbard, C., & Goncalves, V. M. G. (2000). Motor development during the first 6 months: the case of Brazilian infants. *Infant and Child Development*, *3*, 161–166.

157. Santos, D. C. C., Gabbard, C., & Goncalves, V. M. G. (2001). Motor development during the first year: a comparative study. *The Journal of Genetic Psychology*, *162*(2), 143–153. https://doi.org/10.1080/00221320109597956

158. Santos, M. M., Corsi, C., Marques, L. A. P., & Rocha, N. A. C. F. (2013). Comparison of motor and cognitive performance of children attending public and private day care centers. *Brazilian Journal of Physical Therapy*, *17*(6), 579–587. https://doi.org/10.1590/S1413-35552012005000126

159. Santos, I. S., Matijasevich, A., Barros, A. J. D., & Barros, F. C. (2014). Antenatal and postnatal maternal mood symptoms and psychiatric disorders in pre-school children from the 2004 Pelotas Birth Cohort. *Journal of Affective Disorders*, *164*(100), 112–117. https://doi.org/10.1016/j.jad.2014.04.033

160. Santos, N. F., & Costa, R. A. (2015). Parental tobacco consumption and child development. *Jornal de Pediatria*, *91*(4), 366–372. https://doi.org/10.1016/J.JPED.2014.09.006

161. Santos, I. S., Barros, F. C., Munhoz, T., & Matijasevich, A. (2017). Gestational age at birth and behavioral problems from four to 11 years of age: birth cohort study. *BMC Pediatrics*, *17*(1). https://doi.org/10.1186/S12887-017-0936-3

162. Santos, I. S., Blumenberg, C., Munhoz, T. N., Matijasevich, A., Salum, C., Santos Júnior, H. G., dos Santos, L. M., Correia, L. L., de Souza, M. R., Lira, P. I. C., Bortolotto, C. C., Barcelos, R., Altafim, E., Chicaro, M. F., Macana, E. C., & da Silva, R. S. (2024). Maternal depression and child development at 3 years of age: a longitudinal study in a Brazilian child development promotion program. *Pediatric Research*, *95*(4), 1139–1146. https://doi.org/10.1038/S41390-023-02876-9

163. Schmidt, B., Bolze, S. D. A., Vieira, M. L., & Crepaldi, M. A. (2018). Percepções Parentais sobre o Temperamento Infantil e suas Relações com as Variáveis Sociodemográficas das Famílias. *Psicologia: Teoria e Pesquisa*, *34*, e3436. https://doi.org/10.1590/0102.3772E3436

164. Schoen-Ferreira, T. H., De Souza Vitalle, M. S., & Marteleto, M. R. F. (2011). Behavioral problems among children and adolescents with scholastic delay. *Pediatr. Res.*, *69*(4), 367.

165. Shavitt, I., Ayres de Araujo Scatollin, M., Suzart Ungaretti Rossi, A., Pacífico Mercadante, M., Gamez, L., Resegue, R. M., Pisani, L., & do Rosário, M. C. (2022). Transcultural adaptation and psychometric properties of the International Development and Early Learning Assessment (IDELA) in Brazilian pre-school children. *International Journal of Educational Research Open*, *3*, 100138. https://doi.org/10.1016/J.IJEDRO.2022.100138

166. Silva, S. D., Flôres, F. S., Corrêa, S. L., Cordovil, R., & Copetti, F. (2017). Mother’s Perception of Children’s Motor Development in Southern Brazil. *Perceptual and Motor Skills*, *124*(1), 72–85. https://doi.org/10.1177/0031512516676203

167. Silva, M. L., Cavalcante, L. I. C., Heumann, S., & Lima, T. V. R. (2018). Relação entre gênero e desempenho neuropsicomotor de crianças em Belém, Brasil. *Ciência & Saúde Coletiva*, *23*(8), 2721–2730. https://doi.org/10.1590/1413-81232018238.13202016

168. Silva, C. V., Cruz, E. J. S. da, Gouveia, A. J., & Pedroso, J. da S. (2020). Global development and infant language’s assessment in institutional settings. *Early Child Development and Care*, *190*(3), 428–437. https://doi.org/10.1080/03004430.2018.1477774

169. Silva, T. M., Bauer, A., Matijasevich, A., Munhoz, T. N., Barros, A. J. D., Santos, I. S., Tovo-Rodrigues, L., & Murray, J. (2024). Early risk factors for conduct problem trajectories from childhood to adolescence: the 2004 Pelotas (BRAZIL) Birth Cohort. *European Child & Adolescent Psychiatry*, *33*(3), 881–895. https://doi.org/10.1007/S00787-023-02178-9

170. da Silva, M. A. C., de Lucena Martins, C. M., Alencar, M. A., Firmino, P. R. A., & Bandeira, P. F. R. (2025). Association Between Motor Competence, Executive Functions, and Self-Regulation: A Complex Systems Approach. *American Journal of Human Biology*, *37*(8), e70110. https://doi.org/10.1002/AJHB.70110

171. Soares-Marangoni, D. A., Arguelho, A. de O., Mendonça, A. S. G. B., Wiesiolek, C. C., de Lima-Alvarez, C. D., dos Chiquetti, E. M., de Oliveira, E. F., de Medeiros, M. J., Pereira, S. A., & Hasue, R. H. (2025). STORCH Brazil: multicenter cohort study protocol to investigate neurodevelopmental paths and functioning in infants exposed to STORCH in Brazil. *BMC Pediatrics*, *25*(1), 1–13. https://doi.org/10.1186/S12887-025-05548-1/FIGURES/3

172. Solís-Cordero, K., Palombo, C. N. T., Duarte, L. S., Munhoz, R. I., Toriyama, A. T. M., Borges, A. L. V., & Fujimori, E. (2020). Developmental surveillance in primary health care: absence of child development milestones and associated factors. *Revista Brasileira de Saúde Materno Infantil*, *20*(4), 925–934. https://doi.org/10.1590/1806-93042020000400002

173. Sousa, A. F. de, & Rondó, P. H. de C. (2020). *Associação entre fatores maternos e infantis e suspeita de atraso no desenvolvimento neuropsicomotor e alteração comportamental em crianças menores de 24 meses* [Tese, Universidade de São Paulo]. https://doi.org/10.11606/T.6.2020.TDE-28012021-171711

174. Souza, S. C., Leone, C., Takano, O. A., & Moratelli, H. B. (2008). Desenvolvimento de pré-escolares na educação infantil em Cuiabá, Mato Grosso, Brasil. *Cadernos de Saúde Pública*, *24*(8), 1917–1926. https://doi.org/10.1590/S0102-311X2008000800020

175. Stasiak, G. R., Weber, L. N. D., & Tucunduva, C. (2014). Qualidade na Interação Familiar e Estresse Parental e suas Relações com o Autoconceito, Habilidades Sociais e Problemas de Comportamento dos Filhos. *Psico*, *45*(4), 494–501. https://doi.org/10.15448/1980-8623.2014.4.15846

176. Tavares, L. F. F. de A., Landeira-Fernandez, J., & Anunciação, L. (2021). Comparação de dados normativos do “ages and stages Questionnaires” original e adaptado para creches públicas do Rio de Janeiro. *Rev. Psicopedagogia*, *38*(117), 346–363. https://doi.org/10.51207/2179-4057.20210029

177. Teixeira, M. C. T. V., Seraceni, M. F. F., Suriano, R., Sant’ana, N. Z., Carreiro, L. R. R., & Paula, C. S. de. (2014). Fatores de proteção associados a problemas emocionais e comportamentais em escolares. *Estudos de Psicologia (Campinas)*, *31*(4), 539–548. https://doi.org/10.1590/0103-166X2014000400008

178. Tovo-Rodrigues, L., Carpena, M. X., Martins-Silva, T., Santos, I. S., Anselmi, L., Barros, A. J. D., Barros, F. C., Bertoldi, A. D., & Matijasevich, A. (2020). Low neurodevelopmental performance and behavioural/emotional problems at 24 and 48 months in Brazilian children exposed to acetaminophen during foetal development. *Paediatric and Perinatal Epidemiology*, *34*(3), 278–286. https://doi.org/10.1111/PPE.12649

179. Valentini, N. C., & Saccani, R. (2012). Brazilian validation of the Alberta Infant Motor Scale. *Physical Therapy*, *92*(3), 440–447. https://doi.org/10.2522/PTJ.20110036

180. Valentini, N. C., Duarte, M. G., Zanella, L. W., & Nobre, G. C. (2022). Test of Gross Motor Development-3: Item Difficulty and Item Differential Functioning by Gender and Age with Rasch Analysis. *International Journal of Environmental Research and Public Health*, *19*(14). https://doi.org/10.3390/IJERPH19148667

181. Valentini, N. C. (2012). Validity and reliability of the TGMD-2 for Brazilian children. *Journal of Motor Behavior*, *44*(4), 275–280. https://doi.org/10.1080/00222895.2012.700967

182. Venancio, S. I., Buccini, G. S., Alves, C. R. L., Bortoli, M. C., Bernal, R. T. I., Eickmann, S. H., Frias, P. G., Giugliani, E. R. J., & Santos, M. O. (2021). Psychometric properties of the Child Development Assessment Questionnaire (QAD-PIPAS) for use in population studies involving Brazilian children aged 0-59 months. *Jornal de Pediatria*, *97*(6), 637–645. https://doi.org/10.1016/J.JPED.2021.01.003

183. Viegas Da Silva, E., Hartwig, F. P., Barros, F., & Murray, J. (2022). Effectiveness of a large-scale home visiting programme (PIM) on early child development in Brazil: quasi-experimental study nested in a birth cohort. *BMJ Global Health*, *7*(1). https://doi.org/10.1136/BMJGH-2021-007116

184. Viegas da Silva, E., Hartwig, F. P., Santos, T. M., Yousafzai, A., Santos, I. S., Barros, A. J. D., Bertoldi, A. D., da Silveira, M. F., Matijasevich, A., Domingues, M. R., & Murray, J. (2024). Predictors of early child development for screening pregnant women most in need of support in Brazil. *Journal of Global Health*, *14*. https://doi.org/10.7189/JOGH.14.04143

185. Villar, J., Cheikh Ismail, L., Staines Urias, E., Giuliani, F., Ohuma, E. O., Victora, C. G., Papageorghiou, A. T., Altman, D. G., Garza, C., Barros, F. C., Puglia, F., Ochieng, R., Jaffer, Y. A., Noble, J. A., Bertino, E., Purwar, M., Pang, R., Lambert, A., Chumlea, C., … Farhi, F. (2018). The satisfactory growth and development at 2 years of age of the INTERGROWTH-21st Fetal Growth Standards cohort support its appropriateness for constructing international standards. *American Journal of Obstetrics and Gynecology*, *218*(2), S841-S854.e2. https://doi.org/10.1016/j.ajog.2017.11.564

186. Villar, J., Fernandes, M., Purwar, M., Staines-Urias, E., Di Nicola, P., Cheikh Ismail, L., Ochieng, R., Barros, F., Albernaz, E., Victora, C., Kunnawar, N., Temple, S., Giuliani, F., Sandells, T., Carvalho, M., Ohuma, E., Jaffer, Y., Noble, A., Gravett, M., … Kennedy, S. (2019). Neurodevelopmental milestones and associated behaviours are similar among healthy children across diverse geographical locations. *Nature Communications*, *10*(1), 1–10. https://doi.org/10.1038/S41467-018-07983-4;SUBJMETA

187. Wehby, G. L., & McCarthy, A. M. (2013). Economic gradients in early child neurodevelopment: A multi-country study. *Social Science and Medicine*, *78*(1), 86–95. https://doi.org/10.1016/j.socscimed.2012.11.038

188. Zago, A. C., Trettim, J. P., Rubin, B. B., Scholl, C. C., Coelho, F. T., Ulguim, F., de Souza Pinheiro, L. M., de Matos, M. B., Pinheiro, R. T., & de Avila Quevedo, L. (2023). Early motor development: risk factors for delay in a population study in Southern Brazil. *Revista de Saúde Pública*, *57*(1), 59. https://doi.org/10.11606/S1518-8787.2023057004991

189. Zanella, L. W., Valentini, N. C., Copetti, F., & Nobre, G. C. (2021). Peabody Developmental Motor Scales - Second Edition (PDMS-2): Reliability, content and construct validity evidence for Brazilian children. *Research in Developmental Disabilities*, *111*. https://doi.org/10.1016/j.ridd.2021.103871

190. Zuanetti, P. A., Avezum, M. D. M. de M., Ferretti, M. I., Pontes-Fernandes, A. C., Nunes, M. E. N., Liporaci, N. M., Fukuda, M. T. H., & Hamad, A. P. A. (2021). Development of language and arithmetic skills: risk and protective factors. Comparative cross-sectional study. *Sao Paulo Medical Journal*, *139*(3), 210–217. https://doi.org/10.1590/1516-3180.2020.0280.R1.10122020
