# Supplementary material for: Prevalence of Suspected Developmental Delays and Behavioural Problems Identified Using Screening Tools Among Brazilian Children: A Systematic Review and Meta‐Analysis
Source: Child Care Health Dev. 2026 Nov 14;52(6):e70379. doi: 10.1111/cch.70379 (PMC13768938; doi:10.1111/cch.70379)
Supplement: Supplementary file 4 [file CCH-52-e70379-s005.docx]

**Supplementary material 4.** Sensitivity analysis of prevalence of suspected developmental delays among Brazilian children as assessed by screening tools.
